# Heart failure supported by veno-arterial extracorporeal membrane oxygenation (ECMO): a systematic review of pre-clinical models

**DOI:** 10.1186/s40635-020-00303-5

**Published:** 2020-05-25

**Authors:** Silver Heinsar, Sacha Rozencwajg, Jacky Suen, Gianluigi Li Bassi, Maximilian Malfertheiner, Leen Vercaemst, Lars Mikael Broman, Matthieu Schmidt, Alain Combes, Indrek Rätsep, John F. Fraser, Jonathan E. Millar, on behalf of the European Extracorporeal Life Support Organisation (EuroELSO) Innovations Workgroup & the National Health Medical Research Council Australia Centre of Research Excellence for Advanced Cardio-respiratory Therapies Improving Organ Support (NHMRC CREACTIONS)

**Affiliations:** 1 Critical Care Research Group, The Prince Charles Hospital, University of Queensland, Chermside, Brisbane, Australia; 2 Second Department of Intensive Care, North Estonia Medical Centre, Talinn, Estonia; 3 Sorbonne Université, INSERM, UMRS-1166, ICAN Institute of Cardiometabolism and Nutrition, Medical ICU, Pitié-Salpêtrière University Hospital, 47, bd de l’Hôpital, 75651 Paris Cedex 13, France; 4 Department of Internal Medicine II, Cardiology and Pneumology, University Medical Center Regensburg, Regensburg, Germany; 5 Department of Perfusion, University Hospital Gasthuisberg, Louven, Belgium; 6 ECMO Centre Karolinska, Karolinska University Hospital, Stockholm, Sweden; 7 Department of Physiology and Pharmacology, Karolinska Institutet, Stockholm, Sweden; 8 Wellcome-Wolfson Centre for Experimental Medicine, Queen’s University Belfast, Belfast, UK

**Keywords:** Heart failure, Extracorporeal membrane oxygenation, Animal models

## Abstract

**Objectives:**

Veno-arterial extracorporeal membrane oxygenation (VA-ECMO) is increasingly being used to treat patients with refractory severe heart failure. Large animal models are developed to help understand physiology and build translational research projects. In order to better understand those experimental models, we conducted a systematic literature review of animal models combining heart failure and VA-ECMO.

**Studies selection:**

A systematic review was performed using Medline via PubMed, EMBASE, and Web of Science, from January 1996 to January 2019. Animal models combining experimental acute heart failure and ECMO were included. Clinical studies, abstracts, and studies not employing VA-ECMO were excluded.

**Data extraction:**

Following variables were extracted, relating to four key features: (1) study design, (2) animals and their peri-experimental care, (3) heart failure models and characteristics, and (4) ECMO characteristics and management.

**Results:**

Nineteen models of heart failure and VA-ECMO were included in this review. All were performed in large animals, the majority (*n* = 13) in pigs. Acute myocardial infarction (*n* = 11) with left anterior descending coronary ligation (*n* = 9) was the commonest mean of inducing heart failure. Most models employed peripheral VA-ECMO (*n* = 14) with limited reporting.

**Conclusion:**

Among models that combined severe heart failure and VA-ECMO, there is a large heterogeneity in both design and reporting, as well as methods employed for heart failure. There is a need for standardization of reporting and minimum dataset to ensure translational research achieve high-quality standards.

## Introduction

Veno-arterial extracorporeal membrane oxygenation (VA-ECMO) is a therapeutic option for critically ill patients with cardiogenic shock, pulmonary embolism, or septic shock who are refractory to conventional treatments [1–3]. It consists of an extracorporeal life support (ECLS) circuit and a membrane lung with a venous drainage and an arterial return. Advances in technology, miniature ECMO consoles and improved circuit biocompatibility have exponentially increased the use of VA-ECMO over the last decade and helped broaden its indications [4, 5]. To further improve outcomes and reduce complications associated with the use of VA-ECMO, high-quality clinical research is required [6].

Animal models constitute a cornerstone of critical care research, especially in the field of mechanical organ support, as they can provide a basis for understanding physiology and design relevant clinical trials. Although the ultimate goal of animal studies is to reflect the clinical scenario, the variability in methods used sometimes makes it difficult to directly translate the results obtained into clinically valuable therapeutic approaches. Multiple animal models using VA-ECMO have been published over many years; however, a comprehensive comparison between different models, in terms of feasibility and methods, is lacking, causing controversy within the field.

Therefore, we conducted a systematic review to summarize distinctive features of available animal models of heart failure supported by VA-ECMO, and to highlight potential limitations, with the goal of identifying best practices for use in the design of future studies.

## Methods

This systematic review was performed following PRISMA guidelines [7]. The design was prepared in accordance with the SYRCLE guidelines [8], and the protocol was published on the PROSPERO website (https://www2.le.ac.uk/library/find/databases/p/prospero) under the registration number CRD42018090364.

### Inclusion and exclusion criteria

Our review covered animal models of heart failure supported by VA-ECMO with no restriction to the publication language. This comprised studies of all types which matched the following PICO approach: (1) population defined as animals with heart failure; (2) intervention defined as animals treated with VA-ECMO; (3) controls defined as animals not treated with VA-ECMO (when the study involved more than one group); and (4) outcomes comprised data reporting quality, characteristics of heart failure, and ECMO support.

Studies using VA-ECMO in the context of cardiac arrest were excluded, as extracorporeal cardiopulmonary resuscitation (ECPR) represents a different clinical scenario and carries its own definition [9].

### Search strategy and data extraction

We used PubMed, Web of Science, and EMBASE to search for animal models of heart failure on VA-ECMO from January 1st, 1996 to January 1st, 2019. The search contained keywords relevant to cardiac failure and VA extracorporeal membrane oxygenation, applying pre-published animal filters when relevant [10, 11]. References from identified studies and relevant review articles were also searched for additional eligible citations. The full search strategy is provided in the Supplementary materials.

Two independent reviewers (SH and IR) initially screened articles based on their titles and abstracts. Full-text articles were subsequently independently reviewed (SH and SR) and data were extracted according to a data extraction form available in the Supplementary materials eTable 1. In case of discrepancies, an independent reviewer was consulted (JM). We only included data that were presented in the reviewed paper itself, except when the paper relied on a model described elsewhere by the same authors.

### Study outcomes

#### Quality of reporting

Global quality of data reporting was assessed using the ARRIVE guidelines which provide specific recommendations for methodology and results in animal studies (see Supplementary materials eTables 2 and 3) [12].

To assess the methodology used for acute heart failure models, we compared criteria used by each study with established guidelines or large international trials, adapted to fit with animal practice [13–17]. Although not every study was designed to study cardiogenic shock, specifically, we considered it of matter as it is the clinical situation in which VA-ECMO is mostly used. We thus considered that a study had defined cardiogenic shock adequately if (1) it was consistent with the guidelines in force at the time of the experiment; (2) it used a combination of two criteria present in any guidelines including at least one clinical criterion; or (3) it used one criterion present in any guidelines and successfully induced acute heart failure. When a study failed to meet cardiogenic shock criteria, it was considered as “acute heart failure without cardiogenic shock.”

#### Heart failure models: characteristics and comparison

The data extraction protocol consisted of the following parameters: type of heart failure induction, methods used to induce heart failure, and criteria used to define cardiogenic shock (as described above) and complications. Details of the definitions used can be found in the Supplementary materials eTable 1.

#### VA-ECMO support characteristics

Parameters included in the data extraction protocol consisted of the type of console/pump, oxygenator, priming solution, ECMO configuration and access, cannulation technique and size, anticoagulation drug and target. Details of the definitions used can be found in the Supplementary materials eTable 1.

### Statistical analysis

Data were analyzed using descriptive statistics and reported as number of occurrences (percentage) or mean ± standard deviation, unless otherwise stated. Given the heterogeneous nature of included studies and taking into account that the aim of the review is to characterize and assess the quality of the models rather than the study outcomes, no attempt was made at meta-analysis.

## Results

### Study selection and animal characteristics

**Fig. 1:**
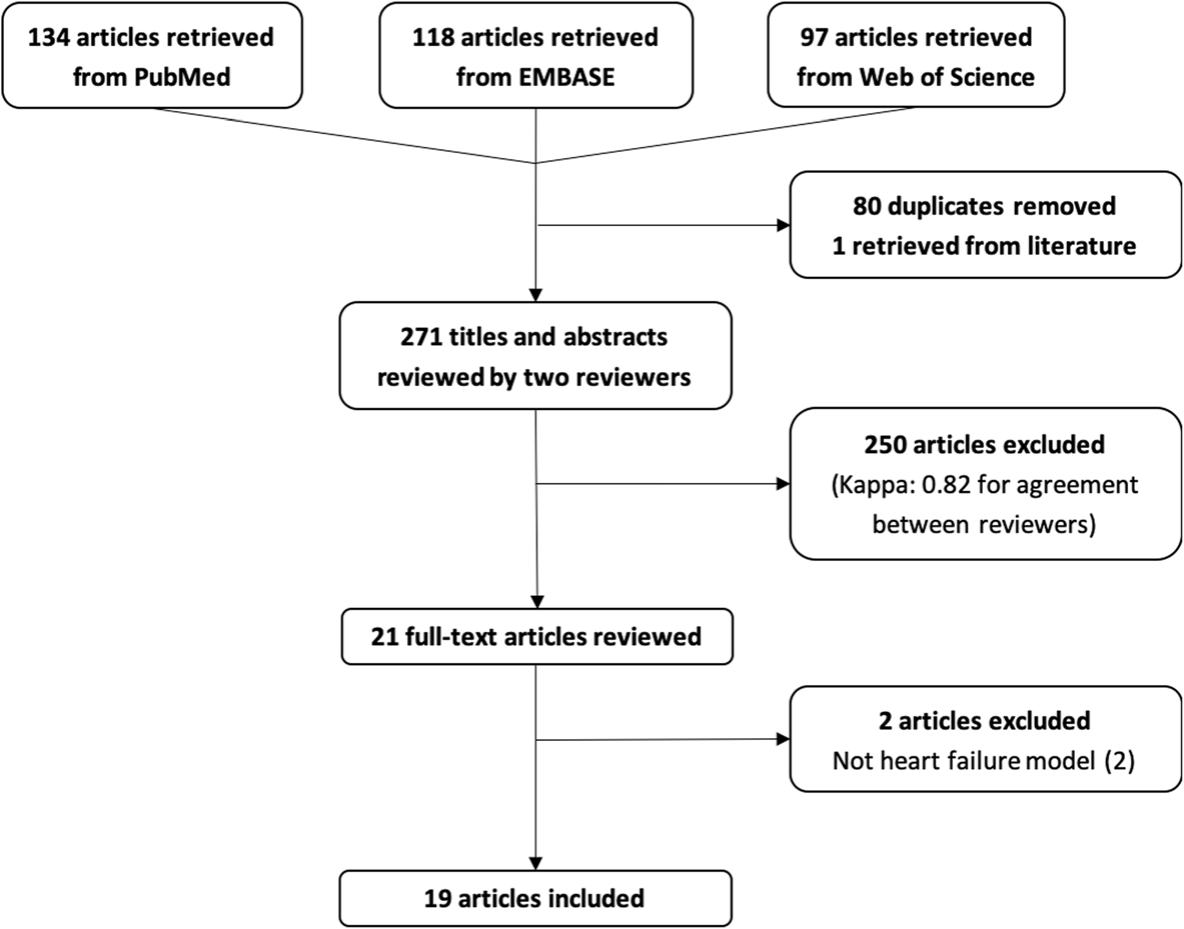
Flow chart of studies selection

A total of 349 articles were retrieved through the search from PubMed, Web of Science, and EMBASE. After removing duplicates, 270 studies were screened by titles and abstracts of which 21 full-text articles were reviewed to finally include 19 studies in the systematic review [18–36] (Fig. 1).

The median study population was ten animals per study (from two to 26) and the majority (12/19, 63%) used porcine models [18, 19]. Animal age was missing in nearly half of the studies reviewed, while anesthetic and airway management were only reported in 22% and 17% of studies, respectively (details can be found in the Supplementary materials eTable 4). Housing and husbandry were systematically omitted, and in 12 out of 19 studies, animals’ fasting protocol was not mentioned. Ten studies (53%) had several groups and could thus be qualified as interventional studies (Table 1).

**Table 1:** Type of studies and main animal characteristics (sorted by animal type)

Study	Year	Species	Study type	Animal age^a^	Number	Heart failure model	ECMO configuration	Group(s)
Sakamoto et al.	2015	Dogs	Other	Adult	21	Myocardial infarction	Vj-Af	ECMO with AMI (*n* = 13) ECMO without cardiac failure (*n* = 8)
Kawashima et al.	2011	Dogs	Physiological	Adult	6	Myocardial infarction	RA-Af	–
Yu et al.	2008	Dogs	Interventional	ND	13	Myocardial infarction	RA-Af	Pulsatile ECMO (*n* = 7) Non-pulsatile ECMO (*n* = 6)
Segesser et al.	2008	Ox	Physiological	ND	5	Pacing	Vf and P^b^ – A_CAR_	–
Møller-Helgestad et al.	2018	Pigs	Interventional	ND	14	Myocardial infarction	Vf-Af	ECMO (*n* = 6) Impella (*n* = 6)
Ostadal et al.	2018	Pigs	Physiological	4-5 months	16	Myocardial hypoxia	Vf-Af	–
Simonsen et al.	2018	Pigs	Interventional	90 days	12	Carbon monoxide poisoning	Vf-Af	ECMO (*n* = 6) Conventional treatment (*n* = 6)
Janak et al.	2017	Pigs	Physiological	4-5 months	8	Myocardial infarction	Vf-Af	–
Vanhuyse et al.	2017	Pigs	Interventional	ND	12	Myocardial infarction	Vf-Af	ECMO + normothermia (*n* = 6) ECMO + hypothermia (*n* = 6)
Esposito et al.	2016	Pigs	Interventional	Adult	10	Myocardial infarction	Vf-Af	ECMO (*n* = 4) TandemHeart (*n* = 4)
Hala et al.	2016	Pigs	Physiological	Up to 6 months	5	Pacing	Vf-Af	–
Itoh et al.	2015	Pigs	Interventional	ND	14	Pacing	RA-AO	Pulsatile ECMO (*n* = 7) Non-pulsatile ECMO (*n* = 7)
Ostadal et al.	2015	Pigs	Physiological	4-5 months	5	Myocardial hypoxia	Vf-Af	–
Brehm et al.	2014	Pigs	Physiological	ND	7	Drug-induced (Esmolol)	Vf-Af	–
Kajimoto et al.	2014	Pigs	Interventional	30-57 days	19	Myocardial infarction	RA-AO	ECMO with AMI (*n* = 6) ECMO with AMI and T3 supplementation (*n* = 6) ECMO without cardiac failure (*n* = 5)
Zhu et al.	2014	Pigs	Interventional	4-5 months	24	Myocardial infarction	Vf-Af	ECMO (*n* = 8) Control/sham (*n* = 8) Drug therapy (*n* = 8)
Bartoli et al.	2013	Pigs	Interventional	ND	47	Myocardial infarction	Vj-A_AO_^c^	ECMO vs IABP (*n* = 10) ECMO vs PFVAD (*n* = 10) ECMO vs CFVAD (*n* = 6)
Sauren et al.	2007	Sheep	Physiological	ND	7	Myocardial infarction	Vf-Af and Vf-AO	–
Naito et al.	2017	Sheep	Physiological	Adult	6	Drug-induced (esmolol)	Vj-A_AO_^c^	–

*AMI* acute myocardial infarction; *Af* femoral artery; *AO* aorta; *AR* right atrium; *asc.* ascending; *CAR* carotid artery; *CFVAD* continuous-flow ventricular assist device; *P* pulmonary artery; *PFVAD* pulsatile-flow ventricular assist device; *Vf* femoral vein; *Vj* jugular vein

^a^Animal age is written as per original paper statement

^b^Venous canula was first inserted into the right atrium through femoral access (as per peripheral VA-ECMO) and then pushed onto the left pulmonary artery; arterial canula was maintained in the carotid throughout the experiment (as per pediatric ECMO configuration)

^c^Arterial canula was inserted surgically directly into the abdominal aorta through a graft

### Quality of reporting

Detailed results regarding the concordance of the applied methodology with the ARRIVE checklist can be found in the Supplementary materials eTables 2 and 3.

General quality of reporting was considered mediocre due to the marginal description of materials and methods and to the heterogeneity in the interventions. As for the description of the methods used to develop heart failure, four studies did not report any criteria to define heart failure [20–23]. One study did not present hemodynamic results, rendering it impossible to assess if the cardiogenic shock was achieved during the experiment, or not [24]. Of the remaining 15 studies, ten (66%) used criteria consistent with adequate cardiogenic shock definition and seven (47%) reported enough data to confirm that animals reached cardiogenic shock (the two Esmolol-induced models and five models of acute myocardial infarction). The last five studies were considered to have reach acute heart failure but without cardiogenic shock (Table 2).

**Table 2:** Criteria used to define cardiogenic shock adapted to animal practice

	Clinical criteria			Hemodynamic criteria		Cardiogenic shock adequately defined?	Cardiogenic shock achieved?
	Arterial hypotension^a^	Pulmonary congestion^b^	End-organ hypoperfusion^c^	Low cardiac output^d^	Elevated filling pressure^e^		
Dogs							
Sakamoto et al.	−	−	−	−	LAP > 10 mmHg	No	N/A
Kawashima et al.	−	−	−	−	−	No	N/A
Yu et al.	No predefined criteria					No	N/A
Ox							
Segesser et al.	“pressure drop”	−	−	−	−	No	N/A
Pigs							
Møller-Helgestad et al.	−	−	SvO_2_ ≤ 35%	**+**	−	**Yes**	**Yes**
Ostadal et al.	−	−	−	**+**	−	**Yes**	**Yes**
Simonsen et al.	−	−	−	**+**	−	**Yes**	**Yes**
Janak et al.	**+**	−	−	**+**	−	**Yes**	No
Vanhuyse et al.	**+**	−	**+**	**+**	−	**Yes**	**Yes**
Esposito et al.	No predefined criteria					No	N/A
Hala et al.	Cardiogenic shock not studied					N/A	
Itoh et al.	No predefined criteria					No	N/A
Ostadal et al.	**+**	−	**+**	−	−	**Yes**	**Yes**
Brehm et al.	**+**	−	−	−	**+**	**Yes**	No
Kajimoto et al.	No predefined criteria					No	N/A
Zhu et al.	**+**	−	−	−	−	**Yes**	No
Bartoli et al.	−	−	Reduction of SvO2 by 10%	**+**	Elevation of LAP ≥ 5 mmHg	**Yes**	**Yes**
Sheep							
Sauren et al.	−	−	−	−	−	No	N/A
Naito et al.	MAP reduction > 20 mmHg	−	−	**+**	LAP increase > 10 mmHg	**Yes**	**Yes**

Data were divided into clinical and hemodynamic variables with “+” indicating the criterion was met and “−” indicating the criterion was not met. When a criterion was correctly defined but met a different threshold, we considered the criterion to be met and wrote the precise threshold used in the study. We considered that a study had defined cardiogenic shock adequately if (i) it was consistent with the guidelines in force at the time of the experiment; (ii) it used a combination of two criteria present in any guidelines including at least one clinical criterion; or (iii) it used one criterion in the context of acute heart failure induction. We considered that a study had achieved cardiogenic shock if those criteria were met during the experiment. Otherwise, it was considered as “acute heart failure without cardiogenic shock”.

*LAP* left atrial pressure; *MAP* mean arterial pressure; *SvO2* venous saturation of oxygen

^a^Systolic blood pressure < 90 mmHg or inotrope, mean arterial pressure (MAP) < 65 mmHg, or > 20% drop in MAP. Based on criteria from SHOCK and IABP-SHOCK II Trial and NICE Clinical Guidelines

^b^Criteria from IABP-SHOCK II trial

^c^Altered mental status, cold/clammy skin and extremities, urine output < 0.5 mL/kg/h, pH < 7.35, elevated serum creatinine, lactate > 2.0 mmol/L. SvO_2_ threshold based on criteria from SHOCK and IABP-SHOCK II Trial, NICE, and ESC Clinical Guidelines

^d^Cardiac index (CI) ≤ 2.2 L/min/m^2^ or cardiac output (CO) < 3.5 L/min or > 20% drop in CO. Based on criteria from SHOCK and IABP-SHOCK II Trials and ESC Clinical Guidelines

^e^Pulmonary capillary wedge pressure (PCWP) ≥ 15 mmHg or increased left atrial pressure (LAP). Based on criteria from SHOCK Trial and ESC Clinical Guidelines

### Heart failure models

#### Characteristics of heart failure models

**Fig. 2:**
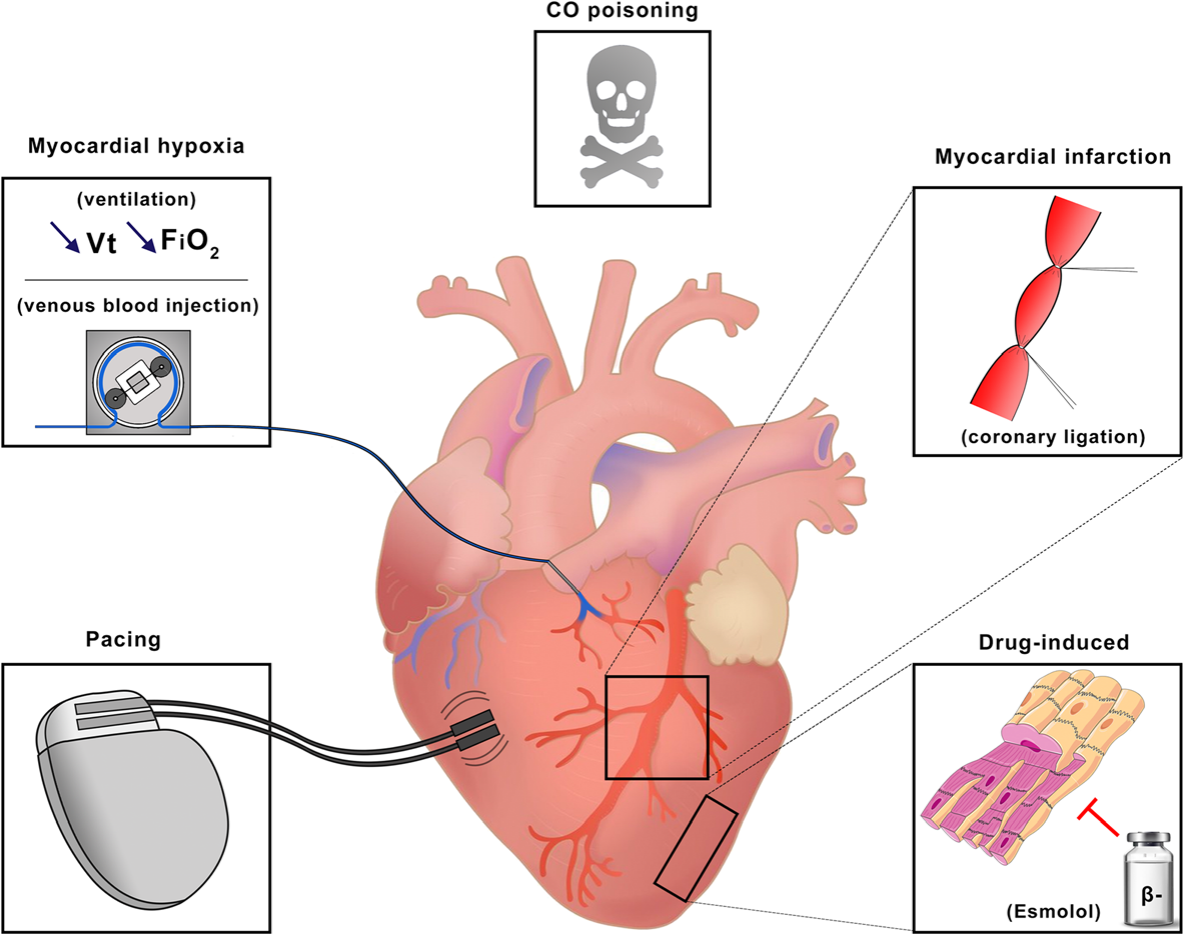
Representation of the five heart failure models that were used in our review. From left to right: ventricular pacing, myocardial hypoxia (through lowering of mechanical ventilation *or* perfusion of desaturated blood in the coronary arteries), CO poisoning, myocardial infarction, and drug-induced heart failure. CO, carbon monoxide; FiO2, inspired fraction of oxygen; Vt, tidal volume. Images were obtained from https://smart.servier.com and are available under a creative commons license

Heart failure models are presented in Fig. 2 and their characteristics are summarized in Table 3. All models but one [25] described acute heart failure. The majority of studies used an acute myocardial infarction (AMI) model (*n* = 11) with left anterior descending (LAD) coronary occlusion, mostly done through ligation. Other models used pacing (*n* = 3) [22, 25, 26] to induce ventricular fibrillation (VF), esmolol infusion (*n* = 2) [27, 28], myocardial hypoxia (*n* = 2) [29, 30], or carbon monoxide poisoning (*n* = 1) [31]. The AMI model was systematically complicated with at least two episodes of irreversible VF leading to death, ranging from 9 to 50% of the subjects. This model seemed to display more complications than the others (no statistical analysis could be made because of poor reporting).

**Table 3:** Detailed characteristics of heart failure model

**Study**	**Heart failure**	**Injury model**	**Procedure description**	**Complications**
Dogs				
**Sakamoto et al.**	Acute	Myocardial infarction	LAD ligation with suture	–
**Kawashima et al.**	Acute	Myocardial infarction	LAD ligation (sequential from distal to proximal every 10 min)	Death from VF (3 out of 6)
**Yu et al.**	Acute	Myocardial infarction	LAD ligation (7 min)	–
Ox				
**Segesser et al.**	Acute	Pacing	External stimulation to induce VF	–
Pigs				
**Møller-Helgestad et al.**	Acute	Myocardial infarction	LMCA injection with alcohol microspheres	Death from VF (2 out of 14)
**Ostadal et al.**	Acute	Myocardial hypoxia	Switch mechanical ventilation to 5 breaths/min, 100 mL *V*_T_, and FiO2 21%	–
**Simonsen et al.**	Acute	Carbon monoxide poisoning	Carbon monoxide administration	Cardiac arrest (6 out of 12) leading to death (*n* = 5)
**Janak et al.**	Acute	Myocardial infarction	LAD and LCx occlusion by balloon inflation (5 min, echo-guided)	–
**Vanhuyse et al.**	Acute	Myocardial infarction	LAD ligation (proximal) with tourniquet (60 min)	–
**Esposito et al.**	Acute	Myocardial infarction	LCx occlusion (proximal) by balloon inflation (30 mins)	Death from VF (2 out of 10)
**Hala et al.**	Chronic*	Pacing	Ventricular pacing (200 bpm)	–
**Itoh et al.**	Acute	Pacing	Direct 3.5 V alternate current to induce VF	–
**Ostadal et al.**	Acute	Myocardial hypoxia	LAD or LCx perfusion with venous blood	VF (2 out of 5)
**Brehm et al.**	Acute	Drug-induced (Esmolol)	Esmolol bolus bolus at 2 mg/kg into the LA	–
**Kajimoto et al.**	Acute	Myocardial infarction	LAD ligation with sutures (10 min)	Death (2 out of 19)
**Zhu et al.**	Acute	Myocardial infarction	LAD ligation between diagonal branches	Death (2 out of 24)
**Bartoli et al.**	Acute	Myocardial infarction	LAD ligation (sequential)	Death from arrhythmias (21 out of 47)
Sheep				
**Sauren et al.**	Acute	Myocardial infarction	LCx (or side branches) ligation	“Unstable” (3 out of 7)
**Naito et al.**	Acute	Drug-induced (Esmolol)	Esmolol bolus at 2 mg/kg into the LA and drip infusion (50 to 500 mg/kg/min)	–

*bpm* beats per minute; *LAD* left anterior descending coronary; *LCx* left circumflex coronary; *LMCA* left main coronary artery; *VF* ventricular fibrillation

^*^A delay of 4 to 8 weeks was respected in order to obtain clinical signs of heart failure

#### VA-ECMO support characteristics

Characteristics of VA-ECMO are summarized in Table 4. Most studies (17/19) employed peripheral or combined cannulation through percutaneous (*n* = 6) or a surgical cut-down (*n* = 4). However, in nine out of 19 studies, cannulation methods were not described. Cannula size was omitted in four studies and none reported the cannula length. Furthermore, and importantly, arterial tip positioning was only confirmed in three [25, 29, 30] out of the twelve studies which used peripheral return cannulation. All studies used intravenous infusion of heparin, yet seven of the 19 studies (37%) did not report any anticoagulation strategy targets. The combination of ECMO consoles, pumps, and oxygenators across studies was highly diverse. Finally, the priming solution was described only in six studies with wide variation [18, 21, 22, 24, 25, 32].

**Table 4:** Detailed characteristics of ECMO support

**Study**	**ECMO type**				**ECMO equipment**			**ECMO settings**	
	**Configuration**	**Cannulation**	**Technique**	**Positioncheck**^**§**^	**Pump**	**Oxygenator**	**Canula size (Fr)**	**Flow**	**ACT target (s)**
Dogs									
**Sakamoto et al.**	Peripheral	Vjr-Afr	ND	No	CBBPX-80	CX-RX15W	ND	Controlled*	ND
**Kawashima et al.**	Combination	RA-Afr	ND	No	Capiox SP-101	ND	28-10	1.5 ± 0.42 L/min	ND
**Yu et al.**	Combination	RA-Af	ND	No	Bio-Source TM200 or T-PLS	ND	21-17	75 mL/kg/min	400-500
Ox									
**Segesser et al.**	Combination	Vf and P – A_CAR_	N/A	N/A	ND	ND	ND	2.5 to 5.6 L/min	> 480
Pigs									
**Møller-Helgestad et al.**	Peripheral	Vfr-Afl	Percutaneous	N/A	ND	ND	ND	3.2 to 4.6 L/min	ND
**Ostadal et al.**	Peripheral	Vf-Af	Percutaneous	Yes	Xenios i-cor	Xenios AG	21-18	Controlled*	200-250
**Simonsen et al.**	Peripheral	Vjr-Afr	Surgical	N/A	Prototype	Maquet Quadrox D	21-15	3500 rpm	ND
**Janak et al.**	Peripheral	Vfl-Afl	Percutaneous	No	Levitronix Centrimag	QUADROX	23-18	Controlled*	210-290
**Vanhuyse et al.**	Peripheral	Vf-Af	Percutaneous	No	Medtronic	Maquet	21-15	ND	180-250
**Esposito et al.**	Peripheral	Vfr-Afr	ND	No	TandemHeart	ND	21-17	Controlled*	300-400
**Hala et al.**	Peripheral	Vf-Af	Percutaneous	**Yes**	Levitronix Centrimag	Maquet Quadrox i	23-18	Controlled*	200-300
**Itoh et al.**	Central	RA-AO	N/A	N/A	HPM-15	ExceLung-prime	16-10	140 mL/kg/min	160-200
**Ostadal et al.**	Peripheral	Vf-Af	Percutaneous	**Yes**	Levitronix Centrimag	Maquet Quadrox i	21-15	Controlled*	180-250
**Brehm et al.**	Peripheral	Vfr-Afr	Surgical	No	Levitronix Centrimag	Maquet Quadrox D	17-19	Controlled*	ND
**Kajimoto et al.**	Central	RA-AO	N/A	N/A	Sarns 8000	CX-RX05RW	ND	80-100 mL/kg/min	ND
**Zhu et al.**	Peripheral	Vfr-Afr	Surgical	No	Biomedicus 550	ND	14-12	ND	180-220
**Bartoli et al.**	Peripheral	Vjr-A_AO_^$^	Surgical	N/A	Not reported	Capiox SX-10	10 to14-18 to 20	0.6-1.16 L/min	> 300
Sheep									
**Sauren et al.**	Combination	Vfl-AO	N/A	N/A	MEDOS DP1	Polystan Safe Maxi Adult	21-18 to 21	2.8 ± 0.9 L/min	> 480
	Peripheral	Vfl-Afl	ND	No					
**Naito et al.**	Peripheral	Vj-A_AO_^$^	ND	N/A	EVAHEART	Biocube 6000	29-21	1.5 ± 0.1 L/min	ND

Brands used for ECMO consoles, pumps and oxygenators (alphabetically): TandemHeart (Cardiac Assist Inc, USA); QUADROX-i Adult, QUADROX-D and Polystan Safe Maxi Adult (Maquet Cardiopulmonary, Germany); MEDOS DP1 (MEDOS, Germany); Medtronic 550 (Medtronic Inc, USA); HPM-15 and ExceLung-prime (MERA, Japan); T-PLS (Twin-Pulse Life Support, SL-1000, New-heartbio Co., Korea); Biocube 6000 (NIPRO, Japan); EVAHEART (Sun Medical Technology Research Corp, Japan); Sarns 8000, CX-RX05RW, CX-RX15W and CAPIOX SX 10 Oxygenator (Terumo, Japan); Levitronix Centrimag (Thoratec, USA); i-cor and Xenios AG (Xenios AG, Germany)

*ACT* activated clotting time; *Af* femoral artery; *Afl* left fermoral artery; *Afr* right fermoral artery; *AO* aorta; *asc.* ascending; *ar* right atrium (in case of percutaneous cannulation); *CAR* carotid artery; *ECMO* extracorporeal membrane oxygenation; *P* pulmonary artery; *RA* right atrium (in case of central cannulation); *rpm* rotation per minutes: *Vf* femoral vein; *Vfl* left femoral vein; *Vfr* right femoral vein; *Vj* jugular vein; *Vjl* left jugular vein; *Vjr* right jugular vein

^$^Arterial canula was inserted surgically directly into the abdominal aorta through a graft

^§^For peripheral canulation, was fluoroscopy or echocardiography used to confirm position of the tip of the canula(s)

^*^ECMO blood flow was a controlled parameter of the experiment

## Discussion

In this systematic review, we provided a comprehensive overview of available pre-clinical models of heart failure supported by VA-ECMO. The main findings of pooled data can be summarized as follows: (1) there was a large heterogeneity in the development of heart failure—AMI model with LAD occlusion was preferentially used and experiments were mostly performed on pigs, (2) materials and methods were poorly reported.

### Main findings

#### Deficiencies in reporting and risks associated

Pre-clinical studies in large animals require consistent and reproducible methods in order to ensure comparability across studies, and ultimately translation into clinical studies. Concerns have been raised regarding the reporting of animal experiments as numerous studies displayed insufficient reporting of methods [37, 38], and our results are in line with those concerns. For example, animals’ characteristics and conditions (e.g., age, feeding management, anesthetic management) may impact animal health or lead to variability in treatment responses [38, 39]. Even more concerning, four studies failed to report the definition of heart failure used in their experiment. It was also found that serious adverse effects, e.g., premature animal death, were poorly described. It should be taken into account that a limited description of adverse effects poses a serious threat to the validity of experimental studies and constitutes substantial bias in post hoc systematic reviews and meta-analyses [40].

#### Heterogeneity in heart failure models

With regard to the development of heart failure, it should be mentioned that the most common indication of VA-ECMO is cardiogenic shock refractory to medical therapy [41]. Thus, to translate animal data to clinical practice, the induced heart failure had to be severe. In our analysis, we used rather broad criteria to define cardiogenic shock, i.e., features described in three different guidelines and a reduction in mean arterial pressure and cardiac output. Irrespective of our wide-ranging criteria, seven out of the 18 studies investigating acute heart failure failed to meet those diagnostic criteria and were considered as “acute heart failure without cardiogenic shock.” Regarding the models used, one should be careful when using the term “acute myocardial infarction” as the methods used behind this term were shown to be variable—from sequential ligation of left circumflex side branches to total proximal irreversible LAD ligation which may impact the severity and predominance of ventricular dysfunction.

#### Heterogeneity in ECMO support

There is a growing consensus that a more accurate terminology is needed in the field of ECLS. As such, it has recently been asserted that “VA-ECMO” should not be applied as an umbrella term for various situations but should be used only to denote the circulatory element of extracorporeal organ support (ECOS) [42]. In the same way, the Extracorporeal Life Support Organization (ELSO, Ann Arbor, MI, USA) has recently published an international multidisciplinary standardized nomenclature for definitions and terminology for ECLS [9].

In our review, we highlighted the poor reporting of, and the lack of a unified terminology for, even very basic data: access (percutaneous versus surgical), priming solution, anticoagulation target, or cannula size.

### Propositions for future studies

#### Choice of animal

Small animals are usually chosen for their accessibility, a lower housing cost, shorter gestation times, and reduced costs for pharmacological treatment, as compared to larger animal models [43]. Even though we could not identify models combining heart failure and ECMO, rodent models supported by ECLS or ECMO have been developed [44–46]. These models should not be abandoned as they can bring preliminary mechanistic results, particularly at cellular or molecular levels, at a lower cost.

Nevertheless, in order to study the effects of VA-ECMO on cardiac failure (especially its physiological impact), considering the currently available technology and the severity of the condition, large animal models are the most adequate. The choice of specific animal species to be used should be based on local resources and laboratory experience. Nevertheless, some specificities are worth mentioning as they might help clinicians and scientists in their choice. In particular, when exploring upper-body blood flow, despite similar cerebral vascularization across different species, the left subclavian artery (LSCA) may be separated from the brachiocephalic trunk at its origin in pigs which may lead to (i) a different arterial curve between left and right upper-body leg, and (ii) a different brain vasoreactivity to laminar flow [47]. Vascular access is also to be mentioned, as sheep femoral arteries form an abrupt angle with the abdominal aorta, thus providing difficult percutaneous access. Finally, ovine and non-human primate models show greater similarity to humans in terms of thrombogenicity mechanisms as compared to dogs or pigs which may impact studies aiming at exploring in vivo impact of ECMO on coagulation [48, 49].

#### Heart failure model and reporting

Cardiogenic shock in humans is mostly caused by AMI or severe myocardial ischemia (anemia, hypoxia); therefore, the most frequently used animal models are developed through coronary artery occlusions [50]. Nevertheless, as found in our study, these models may produce severe and unpredictable adverse events, such as untreatable hemodynamic instability caused by ventricular arrhythmias. In the specific setting of VA-ECMO research, the extent of ischemic injury should be severe yet controllable in order to develop a sustainable cardiac failure, unless extensive and terminal heart failure is being investigated. Up to today, we have found that such models are limited to the use of esmolol [27, 28] and intra-myocardial injection of ethanol [51]—a recently described and promising method for which data still need to be reproduced. Other methods of inducing heart failure have been proposed, in particular, pressure overload models via cardiac banding-debanding (also known as thoracic aortic compression—TAC), leading to successful, precise, and reproducible results in small animals [52, 53]. The aim of these models’ is slightly different as they study the consequences of an “acute on chronic” heart failure. However, they are relevant for the subpopulation of patients which could undergo ECMO, and the characteristics of precision and reproducibility meet the criteria we identified to study the consequences of VA-ECMO. These models would therefore merit further evaluation, as studies on large animals are currently limited [54].

#### VA-ECMO settings and reporting

Unless required by the experiment protocol, we believe VA-ECMO settings and more generally hemodynamic support should be standardized to ensure comparability and translation into clinical studies. A clear definition of cardiogenic shock should be provided, and a strategy to support it (fluid therapy, inotropes, and vasopressors) as well as hemodynamic targets (MAP above 65 mmHg with normalization of arteria lactate) as per current guidelines. Once VA-ECMO support has been started, cannulation and settings should be as standardized as possible as per latest guidelines or practice: femoral percutaneous access with arterial tip position confirmation, 60 mL/kg/min of ECMO blood flow, a membrane fraction of oxygen (F_D_O2) as low as possible in order to reach SaO2 of 92% on the right upper limb, with a sweep gas flow to maintain a stable arterial pH. Ventilation strategy under VA-ECMO is still highly debated, and we do not comment on this since it was not the scope of this review. In Table 5, we propose a minimum dataset based on the latest guidelines [55].

**Table 5:** Proposed minimum reporting dataset for pre-clinical models of heart failure supported by VA-ECMO

Dataset	Example items	Notes/criteria proposed
1. Animal	Species, age, sex, housing and husbandry.	Use ARRIVE guidelines [12]
2. Heart failure model	Method of injury including detailed surgical/medical procedure, timing and delay Heart failure/cardiogenic shock definition Heart failure/cardiogenic shock achievement	Use latest guidelines and/or trials adapted to fit with animal practice
3. Hemodynamic	Hemodynamic targets	MAP > 65 mmHg, arterial lactate < 2 mmol/L *Items mandatory to report*: LVOT VTI, LVEF, aortic valve opening, pulse pressure
	Hemodynamic support strategy	Fluid support (type and volume per kg) and strategy Vasopressor support (type and dose per kg per min) and strategy (first line support, second line support)
4. ECMO type	ECMO configuration Method of cannulation	Peripheral (except in post-cardiotomy setting) Percutaneous femoral access (except in post-cardiotomy setting)
5. ECMO equipment	Pump and oxygenator model Canula model and size Placement confirmation (if peripheral)	Use Maastricht treaty nomenclature [9]
6. ECMO settings	Flow targets Gas exchange targets Anticoagulation treatment and target	60-80 mL/kg/min FmO2 minimal, sweep gas flow to maintain stable pH

*ECMO* extracorporeal membrane oxygenation; *FmO2* membrane fraction of oxygen; *LVEF* left ventricular ejection fraction; *LVOT* left ventricular outflow tract; *MAP* mean arterial pressure; *SvO2* venous saturation of oxygen; *VTI* velocity-time index

### Limitations

Our study has several limitations. Firstly, data extraction into pre-defined categories may result in a simplification of the data presented in the studies reviewed. Secondly, we did not conduct a formal assessment of the risk of bias. Finally, we also excluded studies before 1996 from our analysis and thus, may have excluded viable models.

## Conclusion

In this systematic review, an overview of contemporary animal models of heart failure supported by veno-arterial extracorporeal membrane oxygenation was given. There is a large heterogeneity in methodology for heart failure induction, as well as ECMO management reporting. Future studies should aim at minimizing those reporting failures—most likely through the use of a minimum dataset—in order to standardize these pre-clinical experiments and help better translation to clinical studies.

## Supplementary information

**Additional file 1.**

## Data Availability

All data and materials are available per request.
