# Supplementary material for: Heart failure supported by veno-arterial extracorporeal membrane oxygenation (ECMO): a systematic review of pre-clinical models
Source: Intensive Care Med Exp. 2020 May 25;8:16. doi: 10.1186/s40635-020-00303-5 (PMC7248156; doi:10.1186/s40635-020-00303-5)
Supplement: Supplementary file 1 — Additional file 1. [file 40635_2020_303_MOESM1_ESM.docx]

**Heart failure supported by veno-arterial extracorporeal membrane oxygenation (ECMO): a systematic review of pre-clinical models.**

**Supplemental materials**

**Page 2 PubMed search strategy**

**Page 3 EMBASE search strategy**

**Page 6 Web of Science search strategy**

**Page 8 eTable 1: Data extraction protocol for included variables**

**Page 11 eTable 3: Assessment of methods according to ARRIVE guidelines**

**Page 12 eTable 4: Assessment of results according to ARRIVE guidelines**

**Page 13 eTable 2:** **Anaesthetic and ventilatory management pre-cardiac failure induction**

**Page 14 PRISMA checklist**

**Search strategies**

*PubMed search strategy*: **Use SYRCLE animal filter for PubMed and search**

(((((((((((((((("extracorporeal membrane oxygenation"[MeSH Terms] OR ("extracorporeal"[All Fields] AND "membrane"[All Fields] AND "oxygenation"[All Fields]) OR "extracorporeal membrane oxygenation"[All Fields] OR "ecmo"[All Fields]) OR ("extracorporeal membrane oxygenation"[MeSH Terms] OR ("extracorporeal"[All Fields] AND "membrane"[All Fields] AND "oxygenation"[All Fields]) OR "extracorporeal membrane oxygenation"[All Fields])) OR (venoarterial[All Fields] AND ("extracorporeal membrane oxygenation"[MeSH Terms] OR ("extracorporeal"[All Fields] AND "membrane"[All Fields] AND "oxygenation"[All Fields]) OR "extracorporeal membrane oxygenation"[All Fields]))) OR (veno-arterial[All Fields] AND ("extracorporeal membrane oxygenation"[MeSH Terms] OR ("extracorporeal"[All Fields] AND "membrane"[All Fields] AND "oxygenation"[All Fields]) OR "extracorporeal membrane oxygenation"[All Fields]))) OR (VA[All Fields] AND ("extracorporeal membrane oxygenation"[MeSH Terms] OR ("extracorporeal"[All Fields] AND "membrane"[All Fields] AND "oxygenation"[All Fields]) OR "extracorporeal membrane oxygenation"[All Fields]))) OR (venoarterial[All Fields] AND ("extracorporeal membrane oxygenation"[MeSH Terms] OR ("extracorporeal"[All Fields] AND "membrane"[All Fields] AND "oxygenation"[All Fields]) OR "extracorporeal membrane oxygenation"[All Fields] OR "ecmo"[All Fields]))) OR (veno-arterial[All Fields] AND ("extracorporeal membrane oxygenation"[MeSH Terms] OR ("extracorporeal"[All Fields] AND "membrane"[All Fields] AND "oxygenation"[All Fields]) OR "extracorporeal membrane oxygenation"[All Fields] OR "ecmo"[All Fields]))) OR (VA[All Fields] AND ("extracorporeal membrane oxygenation"[MeSH Terms] OR ("extracorporeal"[All Fields] AND "membrane"[All Fields] AND "oxygenation"[All Fields]) OR "extracorporeal membrane oxygenation"[All Fields] OR "ecmo"[All Fields]))) OR (venoarterial[All Fields] AND ECLS[All Fields])) OR (veno-arterial[All Fields] AND ECLS[All Fields])) OR (VA[All Fields] AND ECLS[All Fields])) OR ECLS[All Fields]) OR ("extracorporeal membrane oxygenation"[MeSH Terms] OR ("extracorporeal"[All Fields] AND "membrane"[All Fields] AND "oxygenation"[All Fields]) OR "extracorporeal membrane oxygenation"[All Fields] OR ("extracorporeal"[All Fields] AND "life"[All Fields] AND "support"[All Fields]) OR "extracorporeal life support"[All Fields])) OR (venoarterial[All Fields] AND ("extracorporeal membrane oxygenation"[MeSH Terms] OR ("extracorporeal"[All Fields] AND "membrane"[All Fields] AND "oxygenation"[All Fields]) OR "extracorporeal membrane oxygenation"[All Fields] OR ("extracorporeal"[All Fields] AND "life"[All Fields] AND "support"[All Fields]) OR "extracorporeal life support"[All Fields]))) OR (veno-arterial[All Fields] AND ("extracorporeal membrane oxygenation"[MeSH Terms] OR ("extracorporeal"[All Fields] AND "membrane"[All Fields] AND "oxygenation"[All Fields]) OR "extracorporeal membrane oxygenation"[All Fields] OR ("extracorporeal"[All Fields] AND "life"[All Fields] AND "support"[All Fields]) OR "extracorporeal life support"[All Fields]))) OR (VA[All Fields] AND ("extracorporeal membrane oxygenation"[MeSH Terms] OR ("extracorporeal"[All Fields] AND "membrane"[All Fields] AND "oxygenation"[All Fields]) OR "extracorporeal membrane oxygenation"[All Fields] OR ("extracorporeal"[All Fields] AND "life"[All Fields] AND "support"[All Fields]) OR "extracorporeal life support"[All Fields]))) AND ((((((("heart failure"[MeSH Terms] OR ("heart"[All Fields] AND "failure"[All Fields]) OR "heart failure"[All Fields] OR ("cardiac"[All Fields] AND "failure"[All Fields]) OR "cardiac failure"[All Fields]) OR ("heart failure"[MeSH Terms] OR ("heart"[All Fields] AND "failure"[All Fields]) OR "heart failure"[All Fields])) OR ("shock, cardiogenic"[MeSH Terms] OR ("shock"[All Fields] AND "cardiogenic"[All Fields]) OR "cardiogenic shock"[All Fields] OR ("cardiogenic"[All Fields] AND "shock"[All Fields]))) OR ("heart failure"[MeSH Terms] OR ("heart"[All Fields] AND "failure"[All Fields]) OR "heart failure"[All Fields] OR ("myocardial"[All Fields] AND "failure"[All Fields]) OR "myocardial failure"[All Fields])) OR (severe[All Fields] AND ("heart failure"[MeSH Terms] OR ("heart"[All Fields] AND "failure"[All Fields]) OR "heart failure"[All Fields] OR ("cardiac"[All Fields] AND "failure"[All Fields]) OR "cardiac failure"[All Fields]))) OR ("heart failure"[MeSH Terms] OR ("heart"[All Fields] AND "failure"[All Fields]) OR "heart failure"[All Fields] OR ("heart"[All Fields] AND "insufficiency"[All Fields]) OR "heart insufficiency"[All Fields])) OR ("heart failure"[MeSH Terms] OR ("heart"[All Fields] AND "failure"[All Fields]) OR "heart failure"[All Fields] OR ("cardiac"[All Fields] AND "insufficiency"[All Fields]) OR "cardiac insufficiency"[All Fields]))

*EMBASE search strategy:* **combine 1., 2. and 3. using AND**

1. 'extracorporeal oxygenation'/exp OR 'venoarterial extracorporeal membrane oxygenation'/exp OR 'venoarterial extracorporeal oxygenation'/exp OR 'extracorporeal life support'/exp OR 'venoarterial extracorporeal life support'/exp OR 'venoarter* ecmo':ti,ab OR 'veno-arter* ecmo':ti,ab OR 'va ecmo':ti,ab OR 'va extracorporeal life support':ti,ab OR 'va ecls':ti,ab OR 'ecls':ti,ab OR 'veno-arter* ecls':ti,ab

2. 'heart failure'/exp OR 'heart failure*':ti,ab OR 'cardia* failure*':ab,ti OR 'myocardial failure*':ti,ab OR 'myocardial decompensat*':ti,ab OR 'cardiogenic shock'/exp OR 'cardiogenic shock'

3. ’animal experiment’/exp OR ’animal model’/exp OR ’experimental animal’/exp OR ’transgenic animal’/exp OR ’male animal’/exp OR ’female animal’/exp OR ’juvenile animal’/exp OR ’animal’:ti,ab OR ’chordata’:ti,ab OR ’vertebrate’:ti,ab OR ’tetrapod’:ti,ab OR ’fish’/exp OR ’amniote’:ti,ab OR ’amphibia’/exp OR ’mammal’:ti,ab OR ’reptile’/exp OR ’sauropsid’/exp OR ’therian’:ti,ab OR ’monotremate’/exp OR ’placental mammals’:ti,ab OR ’marsupial’/exp OR
’Euarchontoglires’:ti,ab OR ’Afrotheria’/exp OR ’Boreoeutheria’/exp OR ’Laurasiatheria’/exp OR ’Xenarthra’/exp OR ’primate’/exp OR ’Dermoptera’/exp OR ’Glires’/exp OR ’Scandentia’/exp OR 'haplorhini':ti,ab OR ’prosimian’/exp OR ’simian’:ti,ab OR ’tarsiiform’/exp OR ’Catarrhini’:ti,ab OR ’Platyrrhini’/exp OR ’ape’:ti,ab OR ’Cercopithecidae’/exp OR ’hominid’:ti,ab OR ’hylobatidae’/exp OR ’chimpanzee’/exp OR ’gorilla’/exp OR ’orangutan’/exp OR ’animal’:ti,ab OR ’animals’:ti,ab OR ’pisces’:ti,ab OR ’fish’:ti,ab OR ’fishes’:ti,ab OR ’catfish’:ti,ab OR ’catfishes’:ti,ab OR ’sheatfish’:ti,ab OR ’silurus’:ti,ab OR ’arius’:ti,ab OR ’heteropneustes’:ti,ab OR ’clarias’:ti,ab OR ’gariepinus’:ti,ab OR ’fathead minnow’:ti,ab OR ’fathead minnows’:ti,ab OR ’pimephales’:ti,ab OR ’promelas’:ti,ab OR ’cichlidae’:ti,ab OR ’trout’:ti,ab OR ’trouts’:ti,ab OR ’char’:ti,ab OR ’chars’:ti,ab
OR ’salvelinus’:ti,ab OR ’salmo’:ti,ab OR ’oncorhynchus’:ti,ab OR ’guppy’:ti,ab OR ’guppies’:ti,ab OR ’millionfish’:ti,ab OR ’poecilia’:ti,ab OR ’goldfish’:ti,ab OR ’goldfishes’:ti,ab OR ’carassius’:ti,ab OR ’auratus’:ti,ab OR ’mullet’:ti,ab OR ’mullets’:ti,ab OR ’mugil’:ti,ab OR ’curema’:ti,ab OR
’shark’:ti,ab OR ’sharks’:ti,ab OR ’cod’:ti,ab OR ’cods’:ti,ab OR ’gadus’:ti,ab OR ’morhua’:ti,ab OR ’carp’:ti,ab OR ’carps’:ti,ab OR ’cyprinus’:ti,ab OR ’carpio’:ti,ab OR ’killifish’:ti,ab OR ’eel’:ti,ab OR ’eels’:ti,ab OR ’anguilla’:ti,ab OR ’zander’:ti,ab OR ’sander’:ti,ab OR ’lucioperca’:ti,ab OR ’stizostedion’:ti,ab OR ’turbot’:ti,ab OR ’turbots’:ti,ab OR ’psetta’:ti,ab OR ’flatfish’:ti,ab OR ’flatfishes’:ti,ab OR ’plaice’:ti,ab OR ’pleuronectes’:ti,ab OR ’platessa’:ti,ab OR ’tilapia’:ti,ab OR ’tilapias’:ti,ab OR ’oreochromis’:ti,ab OR ’sarotherodon’:ti,ab OR ’common sole’:ti,ab OR ’dover sole’:ti,ab OR ’solea’:ti,ab OR ’zebrafish’:ti,ab OR ’zebrafishes’:ti,ab OR ’danio’:ti,ab OR ’rerio’:ti,ab OR ’seabass’:ti,ab OR ’dicentrarchus’:ti,ab OR ’labrax’:ti,ab OR ’morone’:ti,ab OR ’lamprey’:ti,ab OR ’lampreys’:ti,ab OR ’petromyzon’:ti,ab OR ’pumpkinseed’:ti,ab OR ’pumpkinseeds’:ti,ab OR ’lepomis’:ti,ab OR ’gibbosus’:ti,ab OR ’herring’:ti,ab OR ’clupea’:ti,ab OR ’harengus’:ti,ab OR ’amphibia’:ti,ab OR ’amphibian’:ti,ab OR ’amphibians’:ti,ab OR ’anura’:ti,ab OR ’salientia’:ti,ab OR ’frog’:ti,ab OR ’frogs’:ti,ab OR ’rana’:ti,ab OR ’toad’:ti,ab OR ’toads’:ti,ab OR ’bufo’:ti,ab OR ’xenopus’:ti,ab OR ’laevis’:ti,ab OR ’bombina’:ti,ab OR ’epidalea’:ti,ab OR ’calamita’:ti,ab OR ’salamander’:ti,ab OR ’salamanders’:ti,ab OR ’newt’:ti,ab OR ’newts’:ti,ab OR ’triturus’:ti,ab OR ’reptilia’:ti,ab OR ’reptile’:ti,ab OR ’reptiles’:ti,ab OR ’bearded dragon’:ti,ab OR ’pogona’:ti,ab OR ’vitticeps’:ti,ab OR ’iguana’:ti,ab OR ’iguanas’:ti,ab OR ’lizard’:ti,ab OR ’lizards’:ti,ab OR ’anguis fragilis’:ti,ab OR ’turtle’:ti,ab OR ’turtles’:ti,ab OR ’snakes’:ti,ab OR ’snake’:ti,ab OR ’aves’:ti,ab OR ’bird’:ti,ab OR ’birds’:ti,ab OR ’quail’:ti,ab OR ’quails’:ti,ab OR ’coturnix’:ti,ab OR ’bobwhite’:ti,ab OR ’colinus’:ti,ab OR ’virginianus’:ti,ab OR ’poultry’:ti,ab OR ’poultries’:ti,ab OR ’fowl’:ti,ab OR ’fowls’:ti,ab OR ’chicken’:ti,ab OR ’chickens’:ti,ab OR ’gallus’:ti,ab OR ’zebra finch’:ti,ab OR ’taeniopygia’:ti,ab OR ’guttata’:ti,ab OR ’canary’:ti,ab OR ’canaries’:ti,ab OR ’serinus’:ti,ab OR ’canaria’:ti,ab OR ’parakeet’:ti,ab OR ’parakeets’:ti,ab OR ’grasskeet’:ti,ab OR ’parrot’:ti,ab OR ’parrots’:ti,ab OR ’psittacine’:ti,ab OR ’psittacines’:ti,ab OR ’shelduck’:ti,ab OR ’tadorna’:ti,ab OR ’goose’:ti,ab OR ’geese’:ti,ab OR ’branta’:ti,ab OR ’leucopsis’:ti,ab OR ’woodlark’:ti,ab OR ’lullula’:ti,ab OR ’flycatcher’:ti,ab OR ’ficedula’:ti,ab OR ’hypoleuca’:ti,ab OR ’dove’:ti,ab OR ’doves’:ti,ab OR ’geopelia’:ti,ab OR ’cuneata’:ti,ab OR ’duck’:ti,ab OR ’ducks’:ti,ab OR ’greylag’:ti,ab OR ’graylag’:ti,ab OR ’anser’:ti,ab OR ’harrier’:ti,ab OR ’circus pygargus’:ti,ab OR ’red knot’:ti,ab OR ’great knot’:ti,ab OR ’calidris’:ti,ab OR ’canutus’:ti,ab OR ’godwit’:ti,ab OR ’limosa’:ti,ab OR ’lapponica’:ti,ab OR ’meleagris’:ti,ab OR ’gallopavo’:ti,ab OR ’jackdaw’:ti,ab OR ’corvus’:ti,ab OR ’monedula’:ti,ab OR ’ruff’:ti,ab OR ’philomachus’:ti,ab OR ’pugnax’:ti,ab
OR ’lapwing’:ti,ab OR ’peewit’:ti,ab OR ’plover’:ti,ab OR ’vanellus’:ti,ab OR ’swan’:ti,ab OR ’cygnus’:ti,ab OR ’columbianus’:ti,ab OR ’bewickii’:ti,ab OR ’gull’:ti,ab OR ’chroicocephalus’:ti,ab OR ’ridibundus’:ti,ab OR ’albifrons’:ti,ab OR ’great tit’:ti,ab OR ’parus’:ti,ab OR ’aythya’:ti,ab OR
’fuligula’:ti,ab OR ’streptopelia’:ti,ab OR ’risoria’:ti,ab OR ’spoonbill’:ti,ab OR ’platalea’:ti,ab OR ’leucorodia’:ti,ab OR ’blackbird’:ti,ab OR ’turdus’:ti,ab OR ’merula’:ti,ab OR ’blue tit’:ti,ab OR ’cyanistes’:ti,ab OR ’pigeon’:ti,ab OR ’pigeons’:ti,ab OR ’columba’:ti,ab OR ’pintail’:ti,ab OR ’anas’:ti,ab OR ’starling’:ti,ab OR ’sturnus’:ti,ab OR ’owl’:ti,ab OR ’athene noctua’:ti,ab OR ’pochard’:ti,ab OR ’ferina’:ti,ab OR ’cockatiel’:ti,ab OR ’nymphicus’:ti,ab OR ’hollandicus’:ti,ab OR ’skylark’:ti,ab OR ’alauda’:ti,ab OR ’tern’:ti,ab OR ’sterna’:ti,ab OR ’teal’:ti,ab OR ’crecca’:ti,ab OR ’oystercatcher’:ti,ab OR 'haematopus’:ti,ab OR 'ostralegus’:ti,ab OR 'shrew’:ti,ab OR 'shrews’:ti,ab OR 'sorex’:ti,ab OR 'araneus’:ti,ab OR 'crocidura’:ti,ab OR 'russula’:ti,ab OR 'european mole’:ti,ab OR 'talpa’:ti,ab OR 'chiroptera’:ti,ab OR 'bat’:ti,ab OR 'bats’:ti,ab OR 'eptesicus’:ti,ab OR 'serotinus’:ti,ab OR 'myotis’:ti,ab OR 'dasycneme’:ti,ab OR 'daubentonii’:ti,ab OR 'pipistrelle’:ti,ab OR 'pipistrellus’:ti,ab OR 'cat’:ti,ab OR 'cats’:ti,ab OR 'felis’:ti,ab OR 'catus’:ti,ab OR 'feline’:ti,ab OR 'dog’:ti,ab OR 'dogs’:ti,ab OR 'canis’:ti,ab OR 'canine’:ti,ab OR 'canines’:ti,ab OR 'otter’:ti,ab OR 'otters’:ti,ab OR 'lutra’:ti,ab OR 'badger’:ti,ab OR 'badgers’:ti,ab OR 'meles’:ti,ab OR 'fitchew’:ti,ab OR 'fitch’:ti,ab OR 'foumart’:ti,ab OR 'foulmart’:ti,ab OR 'ferrets’:ti,ab OR 'ferret’:ti,ab OR 'polecat’:ti,ab OR 'polecats’:ti,ab OR 'mustela’:ti,ab OR 'putorius’:ti,ab OR 'weasel’:ti,ab OR 'weasels’:ti,ab OR 'fox’:ti,ab OR 'foxes’:ti,ab OR 'vulpes’:ti,ab OR 'common seal’:ti,ab OR 'phoca’:ti,ab OR 'vitulina’:ti,ab OR 'grey seal’:ti,ab OR 'halichoerus’:ti,ab OR 'horse’:ti,ab OR 'horses’:ti,ab OR 'equus’:ti,ab OR 'equine’:ti,ab OR 'equidae’:ti,ab OR 'donkey’:ti,ab OR 'donkeys’:ti,ab OR 'mule’:ti,ab OR 'mules’:ti,ab OR 'pig’:ti,ab OR 'pigs’:ti,ab OR 'swine’:ti,ab OR 'swines’:ti,ab OR 'hog’:ti,ab OR 'hogs’:ti,ab OR 'boar’:ti,ab OR 'boars’:ti,ab OR 'porcine’:ti,ab OR 'piglet’:ti,ab OR 'piglets’:ti,ab OR 'sus’:ti,ab OR 'scrofa’:ti,ab OR 'llama’:ti,ab OR 'llamas’:ti,ab OR 'lama’:ti,ab OR 'glama’:ti,ab OR 'deer’:ti,ab OR 'deers’:ti,ab OR 'cervus’:ti,ab OR 'elaphus’:ti,ab OR 'cow’:ti,ab OR 'cows’:ti,ab OR 'bos taurus’:ti,ab OR 'bos indicus’:ti,ab OR 'bovine’:ti,ab OR 'bull’:ti,ab OR 'bulls’:ti,ab OR 'cattle’:ti,ab OR 'bison’:ti,ab OR 'bisons’:ti,ab OR 'sheep’:ti,ab OR
'sheeps’:ti,ab OR 'ovis aries’:ti,ab OR 'ovine’:ti,ab OR 'lamb’:ti,ab OR 'lambs’:ti,ab OR 'mouflon’:ti,ab OR 'mouflons’:ti,ab OR 'goat’:ti,ab OR 'goats’:ti,ab OR 'capra’:ti,ab OR 'caprine’:ti,ab OR 'chamois’:ti,ab OR 'rupicapra’:ti,ab OR 'leporidae’:ti,ab OR 'lagomorpha’:ti,ab OR 'lagomorph’:ti,ab OR 'rabbit’:ti,ab OR 'rabbits’:ti,ab OR 'oryctolagus’:ti,ab OR ’cuniculus ’:ti,ab OR 'laprine’:ti,ab OR 'hares’:ti,ab OR 'lepus’:ti,ab OR 'rodentia’:ti,ab OR 'rodent’:ti,ab OR 'rodents’:ti,ab OR 'murinae’:ti,ab OR 'mouse’:ti,ab OR 'mice’:ti,ab OR 'mus’:ti,ab OR 'musculus’:ti,ab OR 'murine’:ti,ab OR 'woodmouse’:ti,ab OR 'apodemus’:ti,ab OR 'rat’:ti,ab OR 'rats’:ti,ab OR 'rattus’:ti,ab OR 'norvegicus’:ti,ab OR 'guinea pig’:ti,ab OR 'guinea pigs’:ti,ab OR 'cavia’:ti,ab OR 'porcellus’:ti,ab OR 'hamster’:ti,ab OR 'hamsters’:ti,ab OR 'mesocricetus’:ti,ab OR 'cricetulus’:ti,ab OR 'cricetus’:ti,ab OR 'gerbil’:ti,ab OR 'gerbils’:ti,ab OR 'jird’:ti,ab OR 'jirds’:ti,ab OR 'meriones’:ti,ab OR 'unguiculatus’:ti,ab OR 'jerboa’:ti,ab OR 'jerboas’:ti,ab OR 'jaculus’:ti,ab OR 'chinchilla’:ti,ab OR
'chinchillas’:ti,ab OR 'beaver’:ti,ab OR 'beavers’:ti,ab OR 'castor fiber’:ti,ab OR 'castor canadensis’:ti,ab OR 'sciuridae’:ti,ab OR ’squirrel ’:ti,ab OR 'squirrels’:ti,ab OR 'sciurus’:ti,ab OR 'chipmunk’:ti,ab OR 'chipmunks’:ti,ab OR 'marmot’:ti,ab OR 'marmots’:ti,ab OR 'marmota’:ti,ab OR 'suslik’:ti,ab OR 'susliks’:ti,ab OR 'spermophilus’:ti,ab OR 'cynomys’:ti,ab OR 'cottonrat’:ti,ab OR 'cottonrats’:ti,ab OR 'sigmodon’:ti,ab OR 'vole’:ti,ab OR 'voles’:ti,ab OR 'microtus’:ti,ab OR 'myodes’:ti,ab OR 'glareolus’:ti,ab OR 'primate’:ti,ab OR 'primates’:ti,ab OR 'prosimian’:ti,ab OR 'prosimians’:ti,ab OR 'lemur’:ti,ab OR 'lemurs’:ti,ab OR 'lemuridae’:ti,ab OR 'loris’:ti,ab OR 'bush baby’:ti,ab OR 'bush babies’:ti,ab OR 'bushbaby’:ti,ab OR 'bushbabies’:ti,ab OR 'galago’:ti,ab OR 'galagos’:ti,ab OR 'anthropoidea’:ti,ab OR 'anthropoids’:ti,ab OR 'simian’:ti,ab OR 'simians’:ti,ab OR
'monkey’:ti,ab OR 'monkeys’:ti,ab OR 'marmoset’:ti,ab OR 'marmosets’:ti,ab OR 'callithrix’:ti,ab OR 'cebuella’:ti,ab OR 'tamarin’:ti,ab OR 'tamarins’:ti,ab OR 'saguinus’:ti,ab OR 'leontopithecus’:ti,ab OR 'squirrel monkey’:ti,ab OR 'squirrel monkeys’:ti,ab OR 'saimiri’:ti,ab OR 'night monkey’:ti,ab OR 'night monkeys’:ti,ab OR 'owl monkey’:ti,ab OR 'owl monkeys’:ti,ab OR 'douroucoulis’:ti,ab OR 'aotus’:ti,ab OR 'spider monkey’:ti,ab OR 'spider monkeys’:ti,ab OR 'ateles’:ti,ab OR 'baboon’:ti,ab OR 'baboons’:ti,ab OR 'papio’:ti,ab OR 'rhesus monkey’:ti,ab OR 'macaque’:ti,ab OR 'macaca’:ti,ab OR 'mulatta’:ti,ab OR 'cynomolgus’:ti,ab OR 'fascicularis’:ti,ab OR 'green monkey’:ti,ab OR 'green
monkeys’:ti,ab OR 'chlorocebus’:ti,ab OR 'vervet’:ti,ab OR 'vervets’:ti,ab OR 'pygerythrus’:ti,ab OR 'hominoidea’:ti,ab OR 'ape’:ti,ab OR 'apes’:ti,ab OR 'hylobatidae’:ti,ab OR 'gibbon’:ti,ab OR 'gibbons’:ti,ab OR 'siamang’:ti,ab OR 'siamangs’:ti,ab OR 'nomascus’:ti,ab OR 'symphalangus’:ti,ab OR 'hominidae’:ti,ab OR 'orangutan’:ti,ab OR 'orangutans’:ti,ab OR 'pongo’:ti,ab OR 'chimpanzee’:ti,ab OR 'chimpanzees’:ti,ab OR 'pan troglodytes’:ti,ab OR 'bonobo’:ti,ab OR 'bonobos’:ti,ab OR 'pan paniscus’:ti,ab OR 'gorilla’:ti,ab OR 'gorillas’:ti,ab OR 'troglodytes’:ti,ab NOT 'human*'

*Web of Science search strategy*: **combine 1., 2. and 3. using AND**

1. TS=("cardia* failure" OR "heart failure" OR "myocardia* failure" OR "cardia* insufficiency" OR "cardiogen* shock" OR "heart insufficiency" OR "cardia* decompensat*" OR "myocardia* failure")

2. TS=("extracorporeal membrane oxygen*" OR "extracorporeal oxygen*" OR "venoarterial extracorporeal membrane oxygen*" OR "veno-arterial extracorporeal membrane oxygen*" OR "veno-arterial extracorporeal oxygen*" OR "VA extracorporeal membrane oxygen*" OR "VA extracorporeal oxygen*" OR "VA ECMO" OR "venoarterial ECMO" OR "veno-arterial ECMO" OR ECLS OR "extracorporeal life support" OR "VA ECLS" OR "VA extracorporeal life support" OR "veno-arterial ECLS" OR "venoarterial ECLS" OR "VA extracorporeal life support" OR "veno-arterial extracorporeal life support")

3. TS=("animal experiment" or "animal model" or "experimental animal" or "transgenic animal" or "male animal" or "female animal" or "juvenile animal" OR animal OR chordata OR vertebrate OR tetrapod OR fish OR amniote OR amphibia OR mammal OR reptile OR sauropsid OR therian OR monotremate OR placental mammals OR marsupial OR Euarchontoglires OR Afrotheria OR Boreoeutheria OR Laurasiatheria OR Xenarthra OR primate OR Dermoptera OR Glires OR Scandentia OR Haplorhini OR prosimian OR simian OR tarsiiform OR Catarrhini OR Platyrrhini OR ape OR Cercopithecidae OR hominid OR hylobatidae OR chimpanzee OR gorilla OR orangutan OR animal OR animals OR pisces OR fish OR fishes OR catfish OR catfishes OR sheatfish OR silurus OR arius OR heteropneustes OR clarias OR gariepinus OR fathead minnow OR fathead minnows OR pimephales OR promelas OR cichlidae OR trout OR trouts OR char OR chars OR salvelinus OR salmo OR oncorhynchus OR guppy OR guppies OR millionfish OR poecilia OR goldfish OR goldfishes OR carassius OR auratus OR mullet OR mullets OR mugil OR curema OR shark OR sharks OR cod OR cods OR gadus OR morhua OR carp OR carps OR cyprinus OR carpio OR killifish OR eel OR eels OR anguilla OR zander OR sander OR lucioperca OR stizostedion OR turbot OR turbots OR psetta OR flatfish OR flatfishes OR plaice OR pleuronectes OR platessa OR tilapia OR tilapias OR oreochromis OR sarotherodon OR common sole OR dover sole OR solea OR zebrafish OR zebrafishes OR danio OR rerio OR seabass OR dicentrarchus OR labrax OR morone OR lamprey OR lampreys OR petromyzon OR pumpkinseed OR pumpkinseeds OR lepomis OR gibbosus OR herring OR clupea OR harengus OR amphibia OR amphibian OR amphibians OR anura OR salientia OR frog OR frogs OR rana OR toad OR toads OR bufo OR xenopus OR laevis OR bombina OR epidalea OR calamita OR salamander OR salamanders OR newt OR newts OR triturus OR reptilia OR reptile OR reptiles OR bearded dragon OR pogona OR vitticeps OR iguana OR iguanas OR lizard OR lizards OR anguis fragilis OR turtle OR turtles OR snakes OR snake OR aves OR bird OR birds OR quail OR quails OR coturnix OR bobwhite OR colinus OR virginianus OR poultry OR poultries OR fowl OR fowls OR chicken OR chickens OR gallus OR zebra finch OR taeniopygia OR guttata OR canary OR canaries OR serinus OR canaria OR parakeet OR parakeets OR grasskeet OR parrot OR parrots OR psittacine OR psittacines OR shelduck OR tadorna OR goose OR geese OR branta OR leucopsis OR woodlark OR lullula OR flycatcher OR ficedula OR hypoleuca OR dove OR doves OR geopelia OR cuneata OR duck OR ducks OR greylag OR graylag OR anser OR harrier OR circus pygargus OR red knot OR great knot OR calidris OR canutus OR godwit OR limosa OR lapponica OR meleagris OR gallopavo OR jackdaw OR corvus OR monedula OR ruff OR philomachus OR pugnax OR lapwing OR peewit OR plover OR vanellus OR swan OR cygnus OR columbianus OR bewickii OR gull OR chroicocephalus OR ridibundus OR albifrons OR great tit OR parus OR aythya OR fuligula OR streptopelia OR risoria OR spoonbill OR platalea OR leucorodia OR blackbird OR turdus OR merula OR blue tit OR cyanistes OR pigeon OR pigeons OR columba OR pintail OR anas OR starling OR sturnus OR owl OR athene noctua OR pochard OR ferina OR cockatiel OR nymphicus OR hollandicus OR skylark OR alauda OR tern OR sterna OR teal OR crecca OR oystercatcher OR haematopus OR ostralegus OR shrew OR shrews OR sorex OR araneus OR crocidura OR russula OR european mole OR talpa OR chiroptera OR bat OR bats OR eptesicus OR serotinus OR myotis OR dasycneme OR daubentonii OR pipistrelle OR pipistrellus OR cat OR cats OR felis OR catus OR feline OR dog OR dogs OR canis OR canine OR canines OR otter OR otters OR lutra OR badger OR badgers OR meles OR fitchew OR fitch OR foumart or foulmart OR ferrets OR ferret OR polecat OR polecats OR mustela OR putorius OR weasel OR weasels OR fox OR foxes OR vulpes OR common seal OR phoca OR vitulina OR grey seal OR halichoerus OR horse OR horses OR equus OR equine OR equidae OR donkey OR donkeys OR mule OR mules OR pig OR pigs OR swine OR swines OR hog OR hogs OR boar OR boars OR porcine OR piglet OR piglets OR sus OR scrofa OR llama OR llamas OR lama OR glama OR deer OR deers OR cervus OR elaphus OR cow OR cows OR bostaurus OR bos indicus OR bovine OR bull OR bulls OR cattle OR bison OR bisons OR sheep OR sheeps OR ovis aries OR ovine OR lamb OR lambs OR mouflon OR mouflons OR goat OR goats OR capra OR caprine OR chamois OR rupicapra OR leporidae OR lagomorpha OR lagomorph OR rabbit OR rabbits OR oryctolagus OR cuniculus OR laprine OR hares OR lepus OR rodentia OR rodent OR rodents OR murinae OR mouse OR mice OR mus OR musculus OR murine OR woodmouse ORapodemus OR rat OR rats OR rattus OR norvegicus OR guinea pig OR guinea pigs OR cavia OR porcellus OR hamster OR hamsters OR mesocricetus OR cricetulus OR cricetus OR gerbil OR gerbils OR jird OR jirds OR meriones OR unguiculatus OR jerboa OR jerboas OR jaculus OR chinchilla OR chinchillas OR beaver OR beavers OR castor fiber OR castor canadensis OR sciuridae OR squirrel OR squirrels OR sciurus OR chipmunk OR chipmunks OR marmot OR marmots OR marmota OR suslik OR susliks OR spermophilus OR cynomys OR cottonrat OR cottonrats OR sigmodon OR vole OR voles OR microtus OR myodes OR glareolus OR primate OR primates OR prosimian OR prosimians OR lemur OR lemurs OR lemuridae OR loris OR bush baby OR bush babies OR bushbaby OR bushbabies OR galago OR galagos OR anthropoidea OR anthropoids OR simian OR simians OR monkey OR monkeys OR marmoset OR marmosets OR callithrix OR cebuella OR tamarin OR tamarins OR saguinus OR leontopithecus OR squirrel monkey OR squirrel monkeys OR saimiri OR night monkey OR night monkeys OR owl monkey OR owl monkeys OR douroucoulis OR aotus OR spider monkey OR spider monkeys OR ateles OR baboon OR baboons OR papio OR rhesus monkey OR macaque OR macaca OR mulatta OR cynomolgus OR fascicularis OR green monkey OR green monkeys OR chlorocebus OR vervet OR vervets OR pygerythrus OR hominoidea OR ape OR apes OR hylobatidae OR gibbon OR gibbons OR siamang OR siamangs OR nomascus OR symphalangus OR hominidae OR orangutan OR orangutans OR pongo OR chimpanzee OR chimpanzees OR pantroglodytes OR bonobo OR bonobos OR pan paniscus OR gorilla OR gorillas OR troglodytes NOT human)

**eTable 1: Data extraction protocol for included variables**

| ***Variable*** | | **Categories** | | **Definition of the variable/Comments** | |
| --- | --- | --- | --- | --- | --- |
| **Animal characteristics** | | | | | |
| Number of animals | | Continuous variable | | If only one group: the number of animals included in the total study if only one group. If multiple groups: the number of animals included in the VA-ECMO group. | |
| Species | | Mouse/Rat  Rabbit  Pig  Canine (dog)  Guinea pig  Ovine (sheep, goat)  Other  More than one | | Animal species used in the experiment | |
| Sex | | Male  Female  Both  Not reported | | Sex of the animals used in the experiment. | |
| Weight | | Weight in kgs  (continuous variable) | | Weight of the animal reported in kilograms. | |
| Age | | Pediatric  Adult  Both  Not reported | | Age of the animals used in the experiment. If the authors clearly state that the model is pediatric or neonatal then pick “pediatric”. If not, then pick “adult”. | |
| **Anesthesia, ventilation and monitoring pre-ECMO** | | | | | |
| Fasted before experiments | | Yes  No  Not reported | | Report if the animals fasted overnight before the experiment. | |
| Induction of anesthesia | | Inhalation  Intravenous  Intramuscular  Intraperitoneal  Combination | | The type of anesthesia used for induction. If a combination is used or if groups results in different anesthesia strategies being compared, choose “combination”.  The induction is the first part where the animal is awake and anesthesia is given in order to be able to intubate the animal. | |
| Maintenance of anesthesia | | Inhalation  Intravenous  Intramuscular  Intraperitoneal  Combination  Not reported | | The type of anesthesia used for maintenance. If a combination is used or if groups results in different anesthesia strategies being compared, choose “combination”.  The maintenance is the second part where anesthesia is maintained throughout the experiment. | |
| Airway | | Endotracheal tube  Tracheostomy  Not reported | | The type of airway accessed for ventilation support throughout the experiment. | |
| Standard monitoring | | Arterial blood pressure  Arterial blood gases  Invasive arterial blood pressure + arterial blood gases | | Blood pressure can be monitored non-invasively or by insertion of an arterial line into an artery.  Arterial blood samples can be drawn from the arterial line and analyzed for e.g. pH, lactate, PaO_2_, PaCO_2_. | |
| Rest period | | Yes  No  Not reported | | A period following instrumentation where animals are allowed to rest before the actual experiment is started. | |
| **Heart failure characteristics** | | | | | |
| Category of heart failure induction | | Pacing  Myocardial infarction  Drug-induced  Hypoxia  Combination  Other | | Method by which heart failure is induced. | |
| Method description for heart failure induction | | String | | Precise the clear method used to induce heart failure. When describing the procedure, be as specific as the authors were (eg.ligation of the main artery for 10 minutes with sutures). | |
| Complications due to heart failure | | Arrhythmia  Cardiogenic shock  Death  Other  None | | Complications during or following heart failure induction. The most common complications are arrhythmia (eg atrial fibrillation, ventricular fibrillation) or cardiogenic shock (defined by the use of vasopressors/inotropes to maintain a mean arterial pressure above 65 mmHg) or death. Only report cardiogenic shock as a complication if not intended in the study. In case of death, precise the cause when possible. | |
| Clear criteria for definition of heart failure | | Yes  No | | Has the author clearly defined criteria for when cardiac arrest is declared. For example, did animal hemodynamic parameters show a significant decrease in mean arterial pressure or left ventricular ejection fraction? | |
| Heart failure definition consistent with the ESC guidelines. | | Yes  No  N/A | | Was cardiac failure definition consistent with 2016 ESC Guidelines for the diagnosis and treatment of acute and chronic heart failure, as follows;  1) Sings and symptoms of heart failure;  2) Reduced LVEF (<50%);  3) If LVEF >40%, also:   1. Elevetated levels of natriuretic peptides; 2. At least one additional criterion:    1. relevant structural heart disease (left ventricular hypertophy an/or left atrial enlargement);    2. diastolic dysfunction. | |
| Cardiac function as endpoint | | Yes  No | | Was cardiac function one of the endpoint for the experiment. If yes, how was it evaluated (eg cardiac output, left ventricular ejection fraction, dpdtmax/dpdtmin…) | |
| **VA-ECMO characteristics** | | | | | |
| ECMO pump | | String | | The type of console/pump used for ECMO set up (eg Levitronix Centrimag, Medos DP1…) | |
| Oxygenator | | String | | The type of oxygenator used for ECMO set up (eg Quadrox-i…) | |
| Priming solution | | String  + Heparin (Yes/No) | | The type of solution used for ECMO priming (eg normal saline, ringer’s lactate) and use of heparin or not in the solution. | |
| ECMO configuration | | Peripheral  Central  Combination | | The type of ECMO configuration used.  A peripheral configuration is defined by the use of a femoral/sub-clavian (adult) or a carotid (pediatric) arterial return whereas a central configuration is defined by the use of an ascending aorta return. Venous canula does not define the type of configuration. | |
| Venous access | | Jugular  Femoral  Right atrium  Other | | The site used for venous inflow access. Main sites include internal jugular vein (jugular), femoral vein (femoral) and right atrium. | |
| Arterial access | | Carotid  Femoral  Ascending aorta  Other | | The site used for arterial outflow access. Main sites include carotid, femoral artery (femoral) and ascending aorta. | |
| Canulation technique | | Percutaneous  Surgical  Not reported | | The technique used for canulation. The two techniques are either percutaneous access (Seldinger technique) or surgical cut-down. | |
| Canula size | | Continuous variable (in French) | | The size of both venous and arterial canula used. | |
| Cannula length | | Continuous variable (in centimeters) | | The length of both venous and arterial canula used. | |
| Position check | | Yes  Not reported | | For peripheral canulation, was fluoroscopy or echocardiography used to confirm position of the tip of the canula(s). | |
| Anticoagulation | | Heparin  Bivilarubin  Other | | The type of anticoagulation used throughout the experiment. Most studies use unfractioned heparin (UFH) as a standard for anticoagulation. | |
| ACT target | | Continuous variable (in seconds) | | The target for anticoagulation monitoring as reported by the ACT. | |
| **Other** | | | | | |
| Type of study | | Interventional  Physiological  Combination  Other | | What is the purpose of study? Is it to test a new intervention or compare two interventions and to see which one improves outcome (interventional study) or is the purpose of the study of a more investigative/ mechanistic character for studying the physiology of VA-ECMO support (physiological) ? | |
| Refer to other article regarding methodology | | Yes  No | | Whether or not the article is referring to another study in regard to the methodology used. | |

**eTable 2: Assessment of methods according to ARRIVE guidelines checklist [1]**

| **Study** | **Ethics** | **Study design** | | | **Procedures** | | | | **Animals** | | **Sample size** | | | **Allocation** | | **Outcomes** | **Statistics** |
| --- | --- | --- | --- | --- | --- | --- | --- | --- | --- | --- | --- | --- | --- | --- | --- | --- | --- |
|  |  | **groups** | **biais minimisation** | **exp. unit** | **how** | **when** | **where** | **why** | **species, strain, sex** | **housing** | **number** | **calculation** | **allocation** | | **order** |  |  |
| Sakamoto et al | 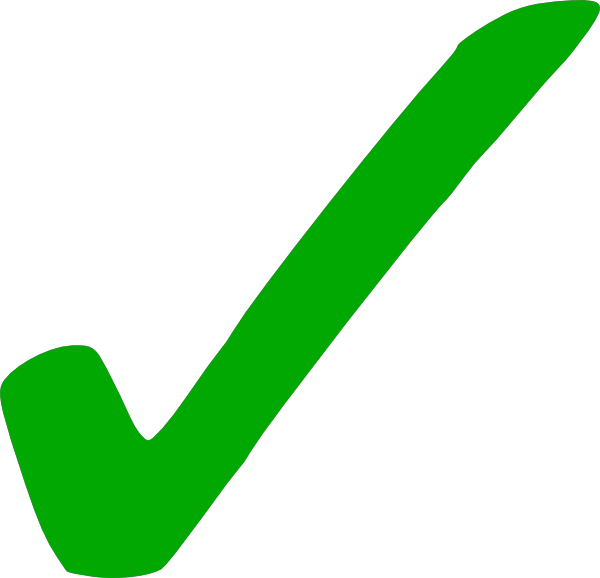 | 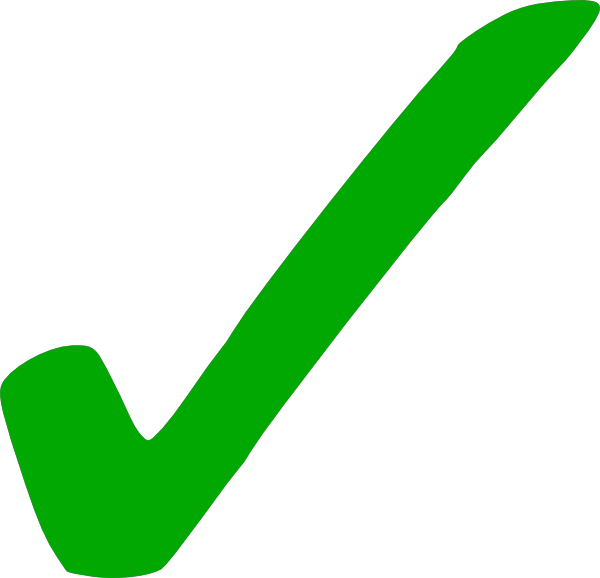 | ❌ | 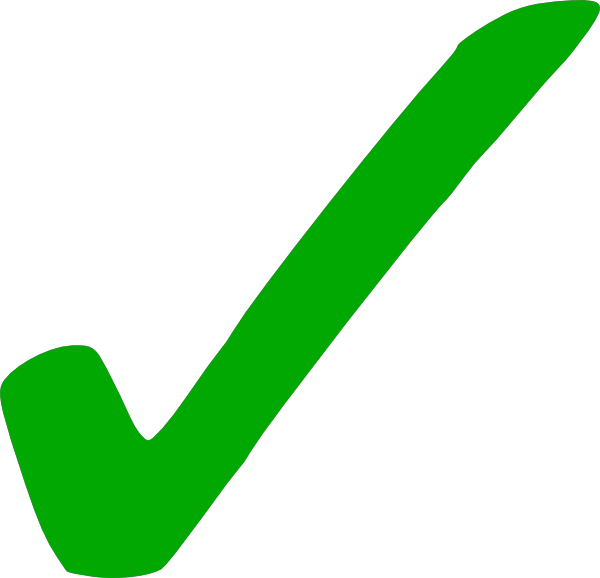 | 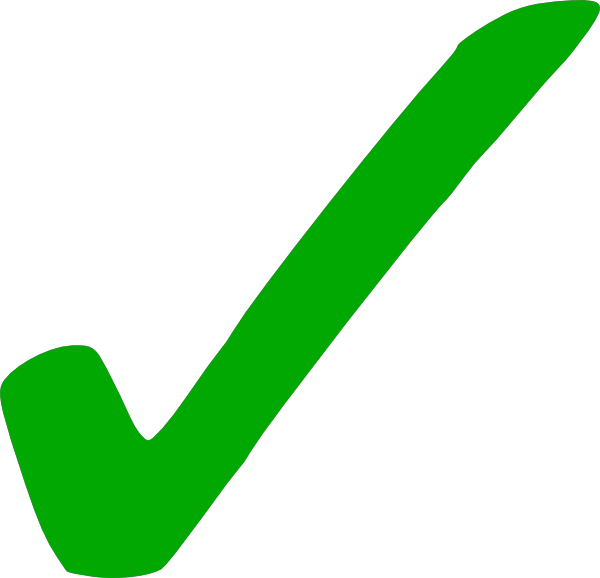 | ❌ | ❌ | ❌ | ❌ | ❌ | 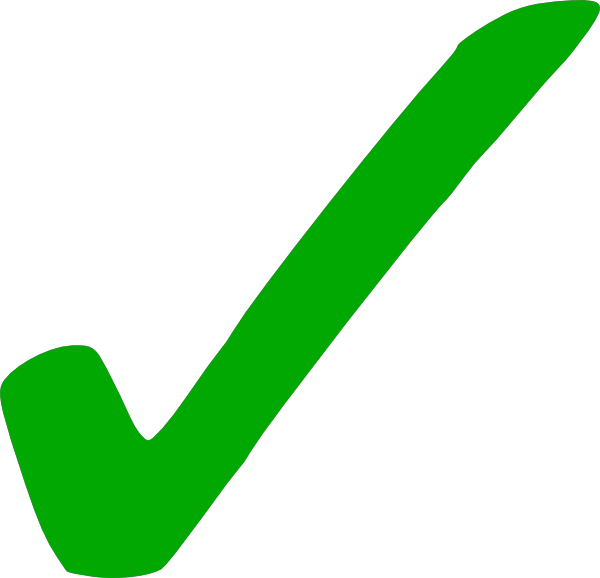 | ❌ | ❌ | | 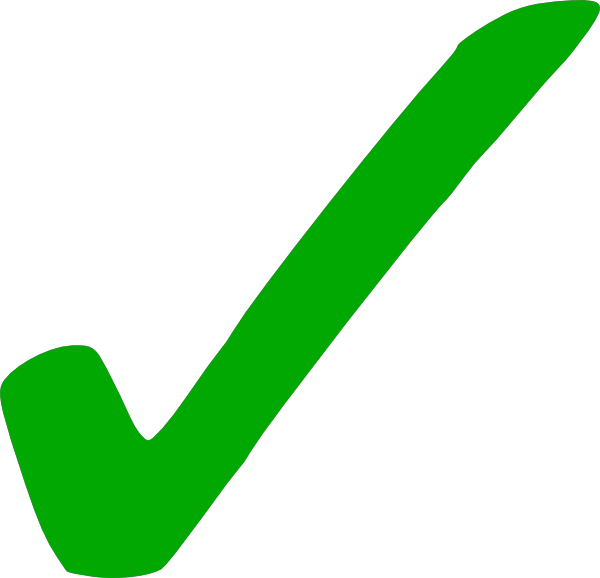 | +/- | 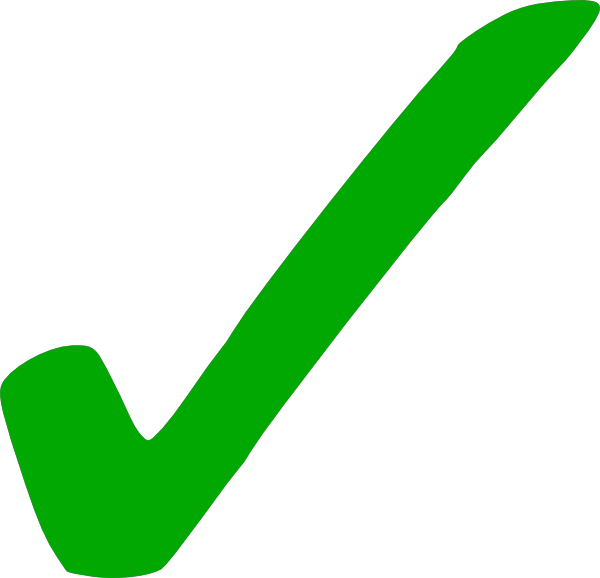 |
| Kawashima et al | 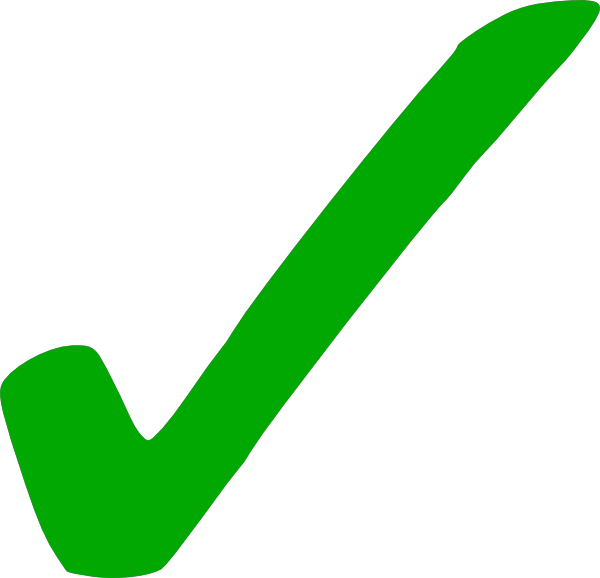 | 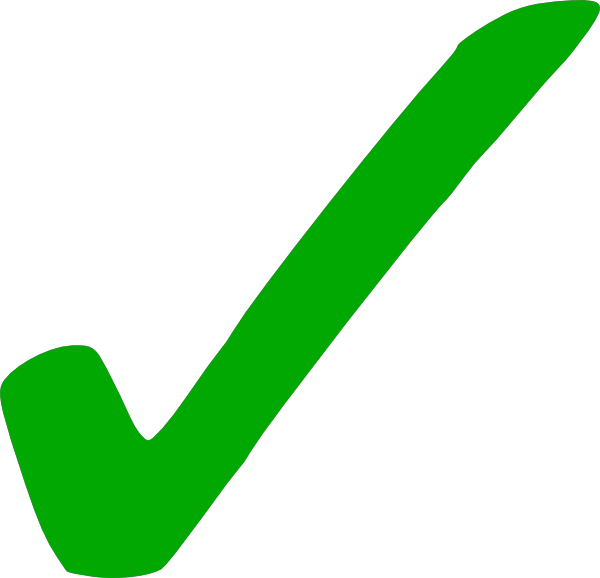 | 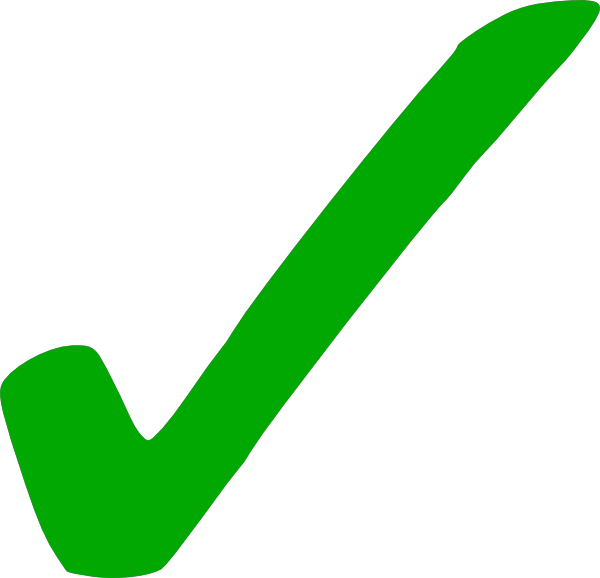 | 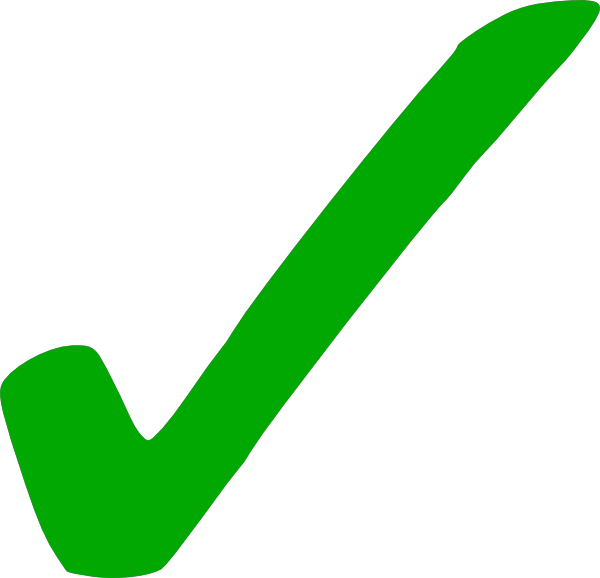 | ❌ | ❌ | ❌ | ❌ | ❌ | ❌ | 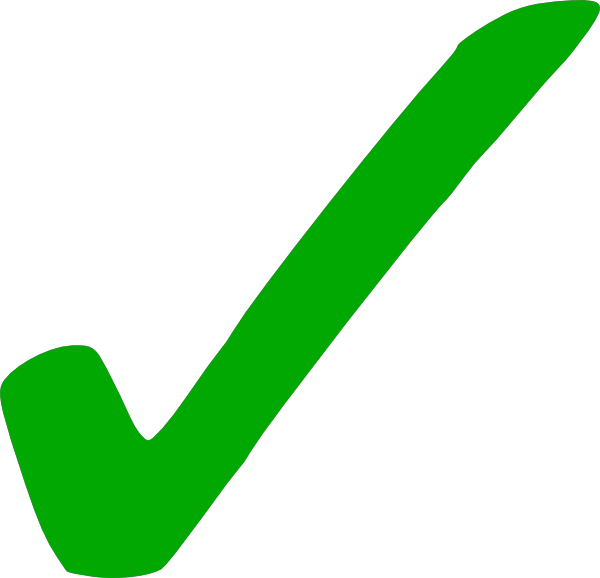 | ❌ | N/A | | | +/- | 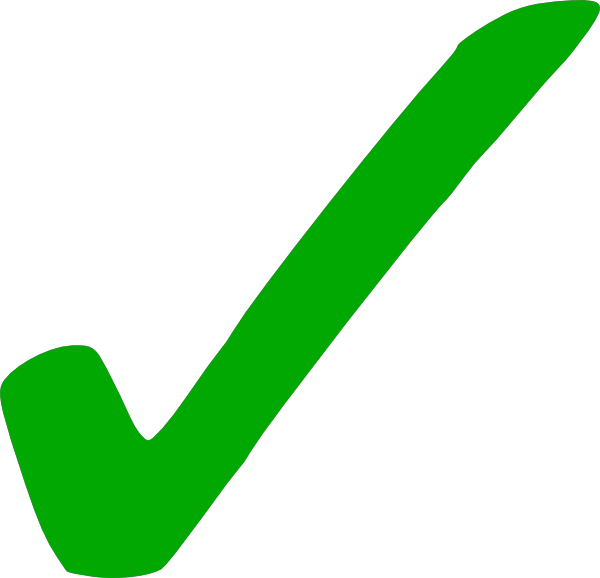 |
| Yu et al | +/- | 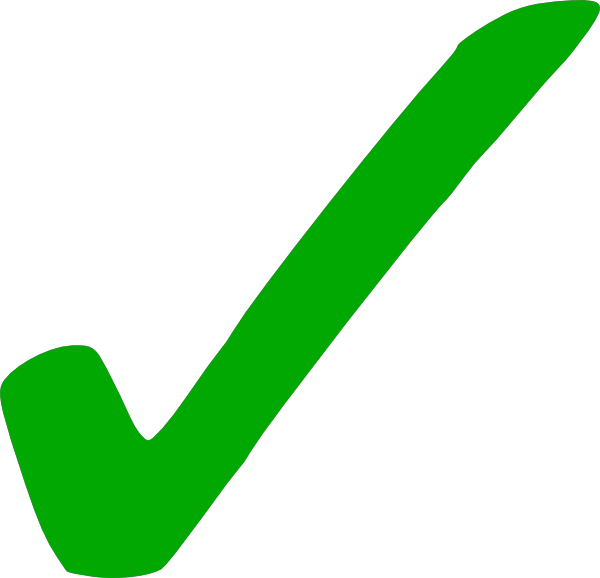 | 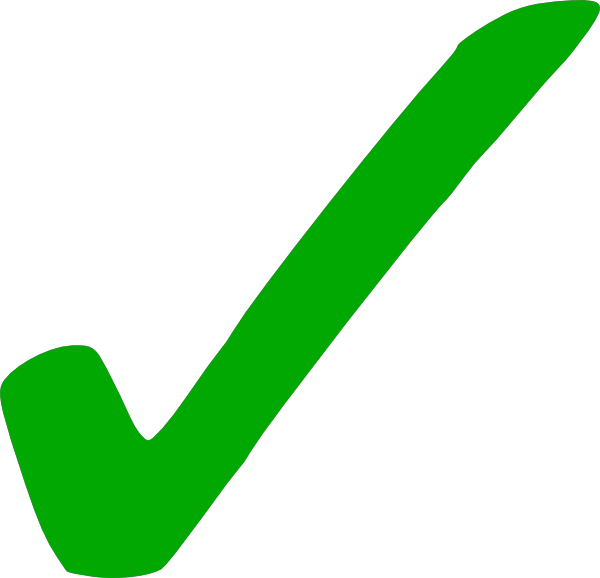 | 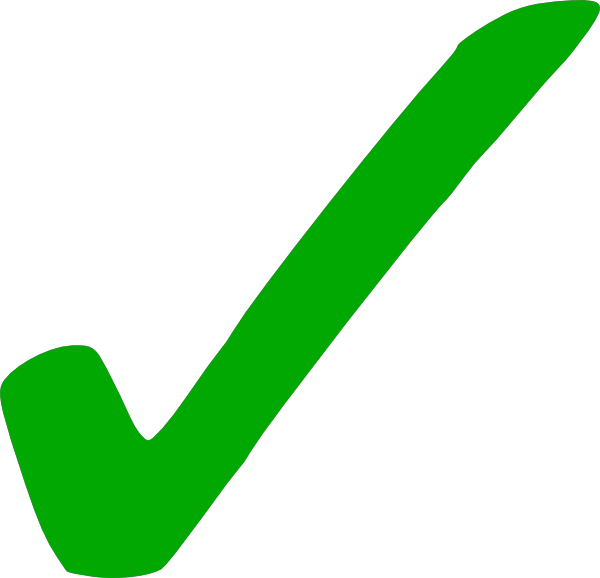 | 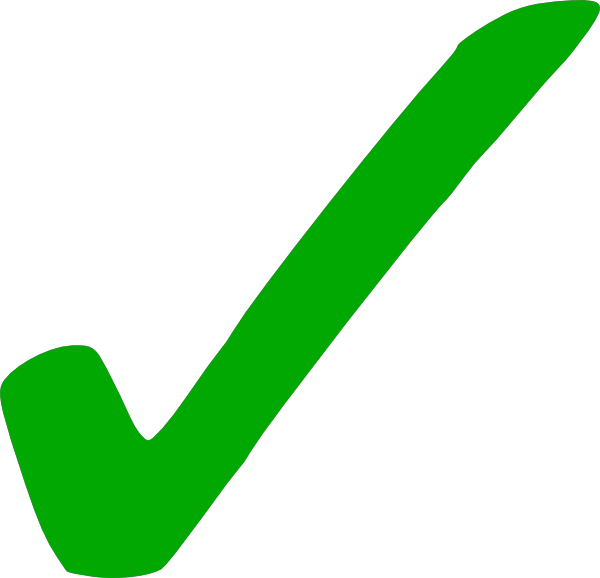 | ❌ | ❌ | ❌ | ❌ | ❌ | 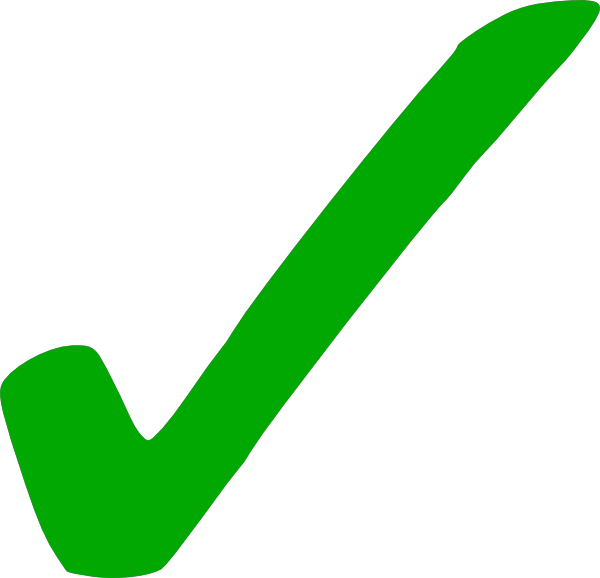 | ❌ | 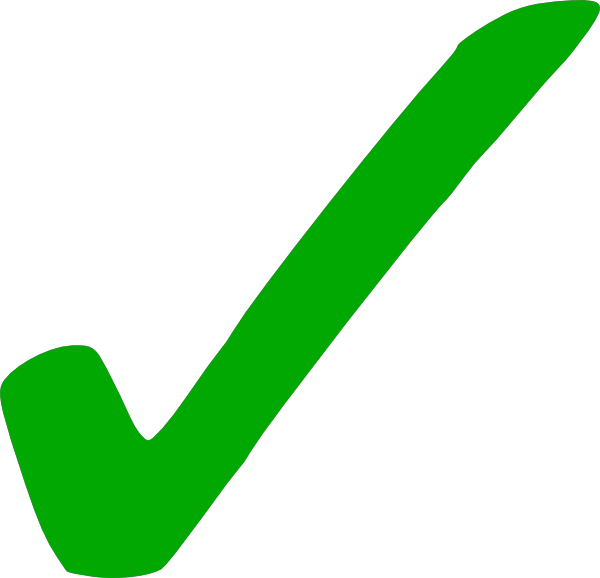 | | +/- | +/- | 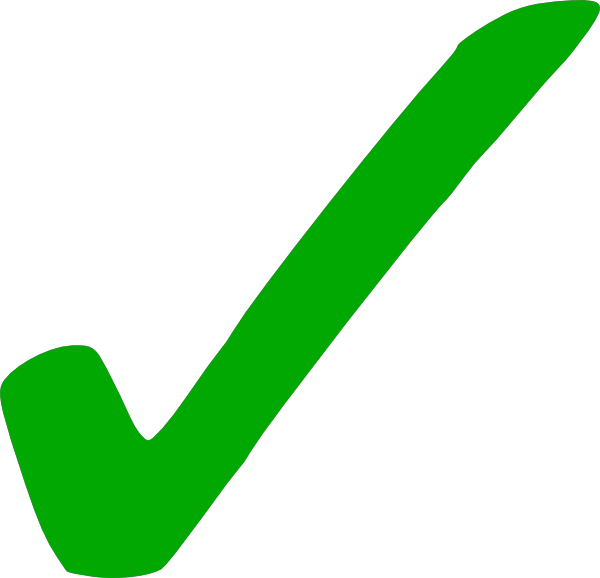 |
| Segesser et al | +/- | 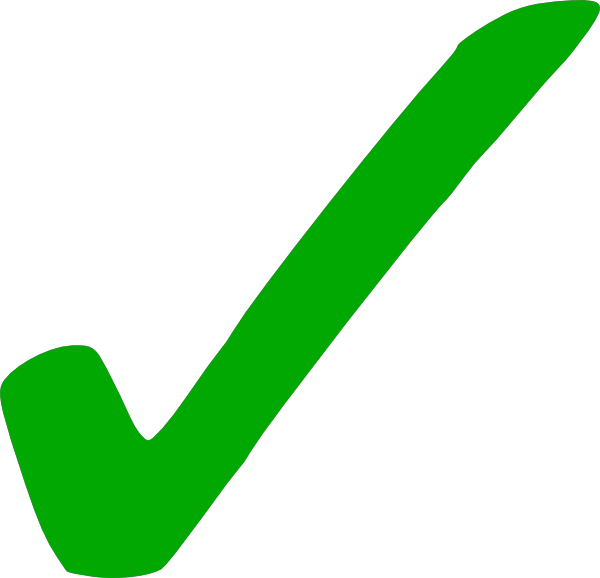 | ❌ | 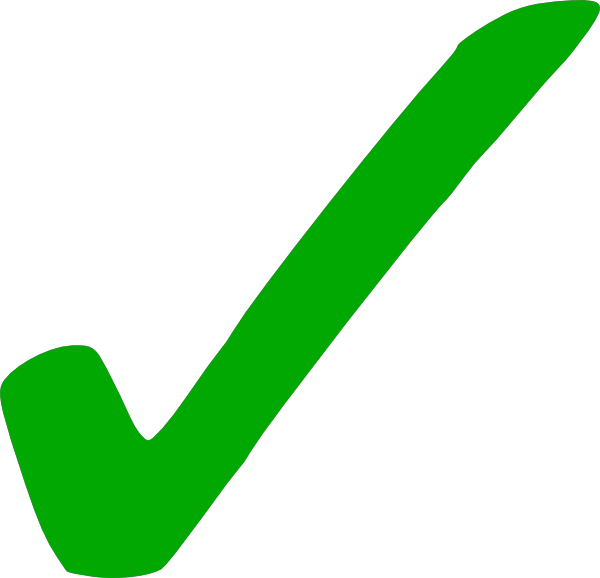 | +/- | ❌ | ❌ | ❌ | +/- | ❌ | 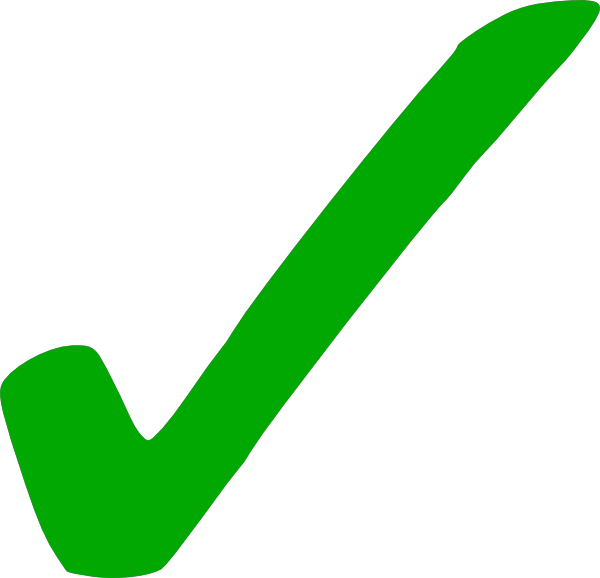 | ❌ | N/A | | | +/- | 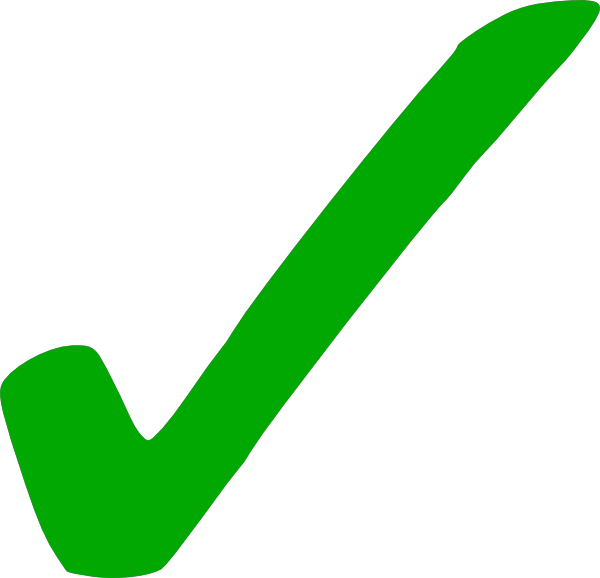 |
| Møller-Helgestad et al | 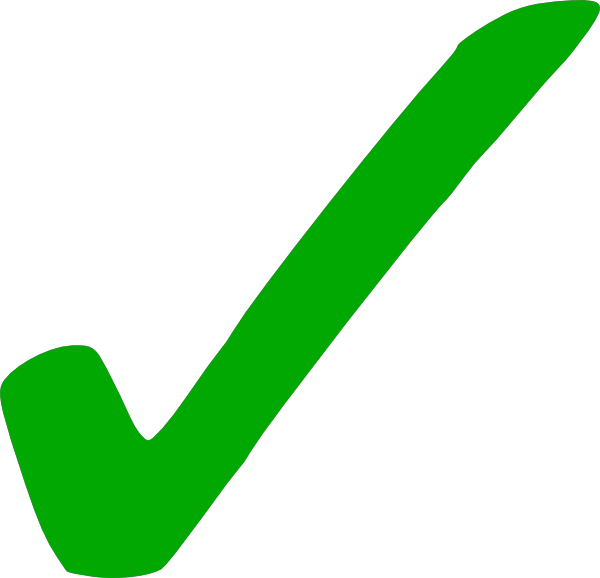 | 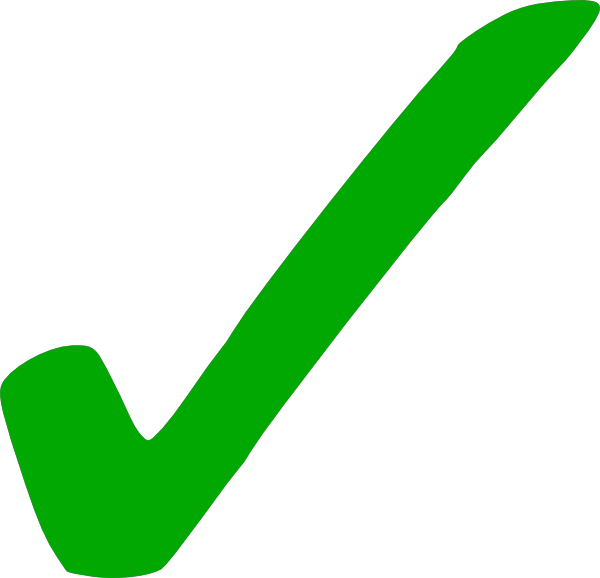 | ❌ | 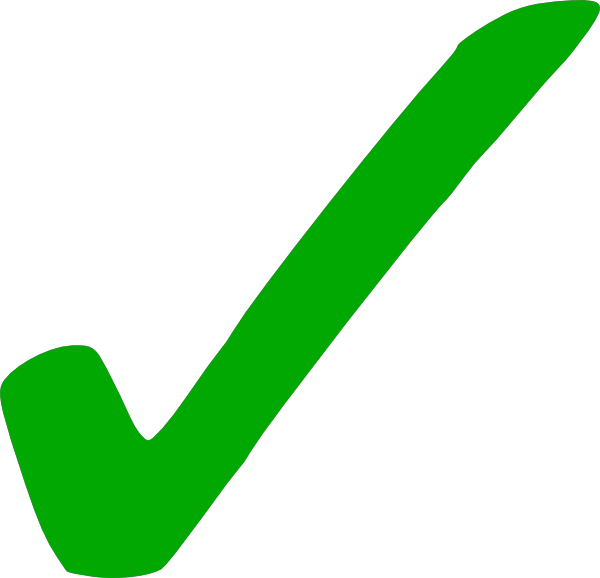 | 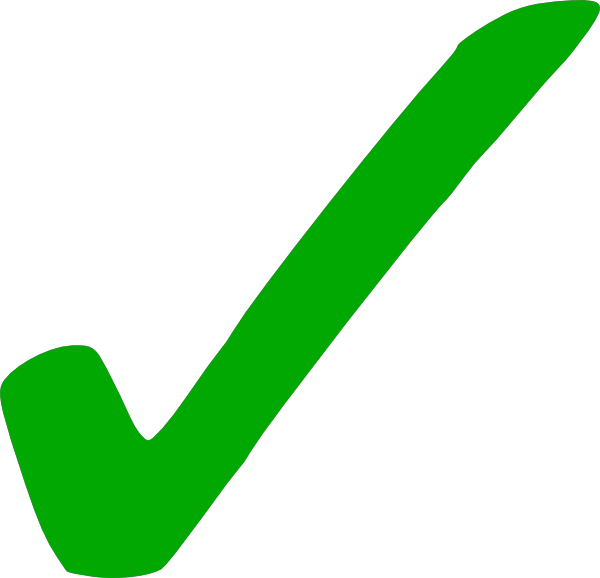 | ❌ | ❌ | ❌ | 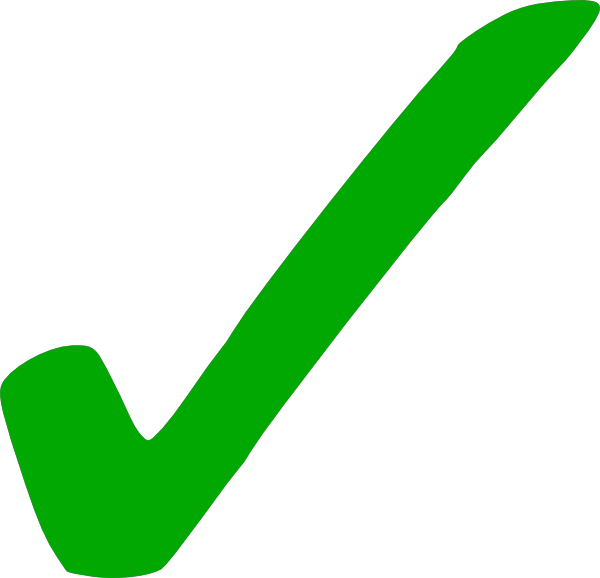 | ❌ | 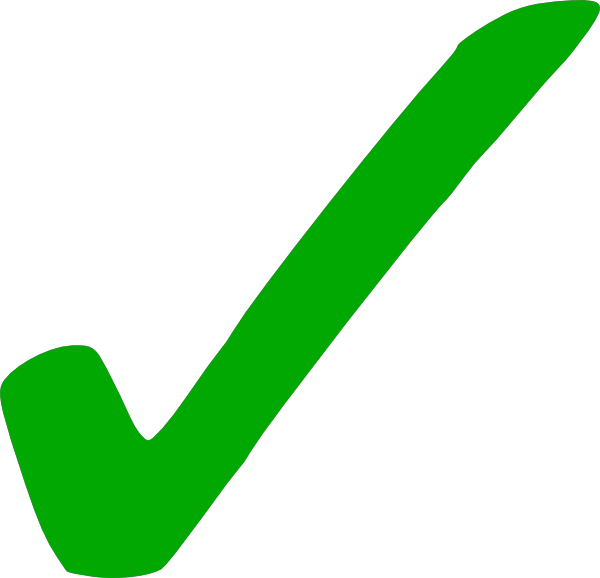 | ❌ | ❌ | | 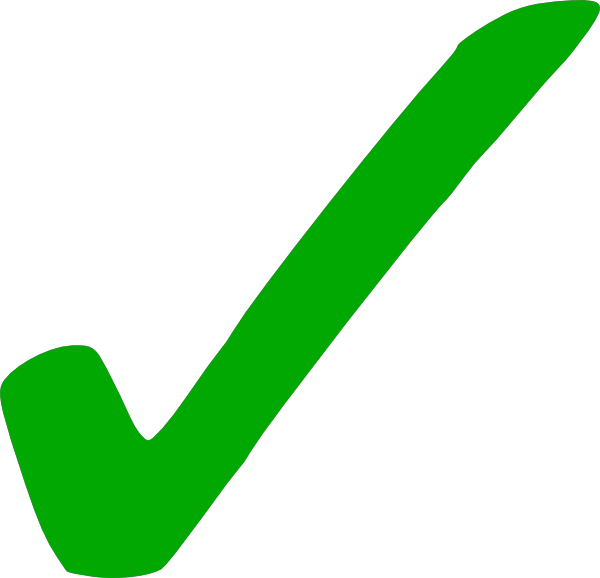 | 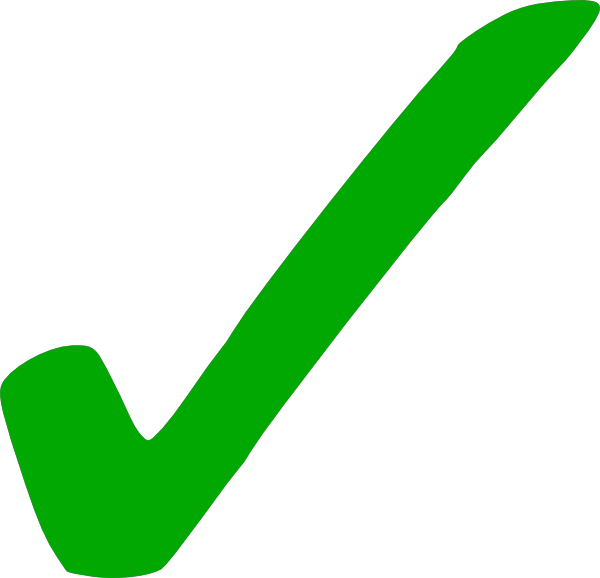 | 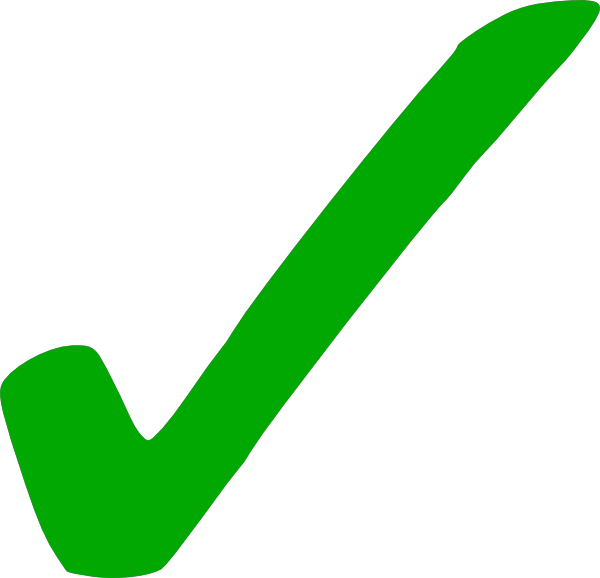 |
| Ostadal et al | 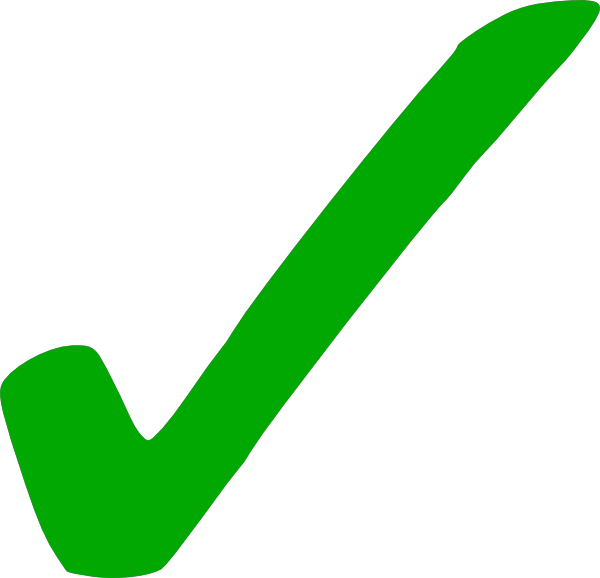 | 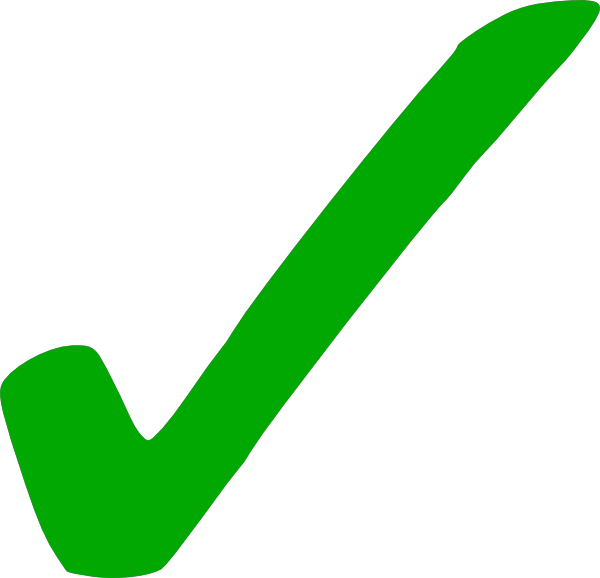 | 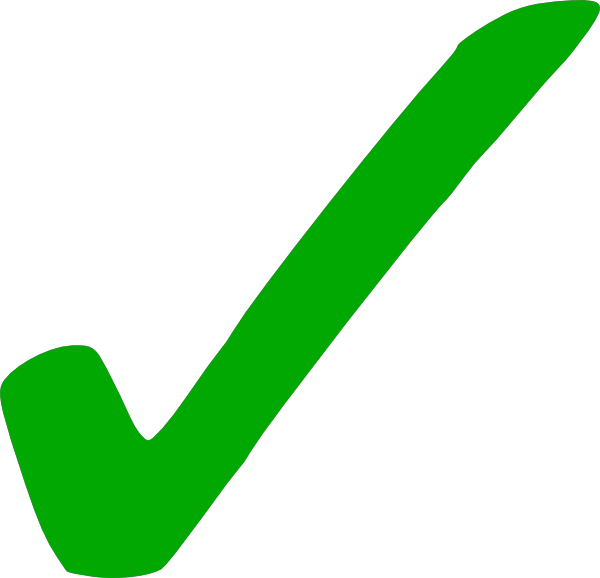 | 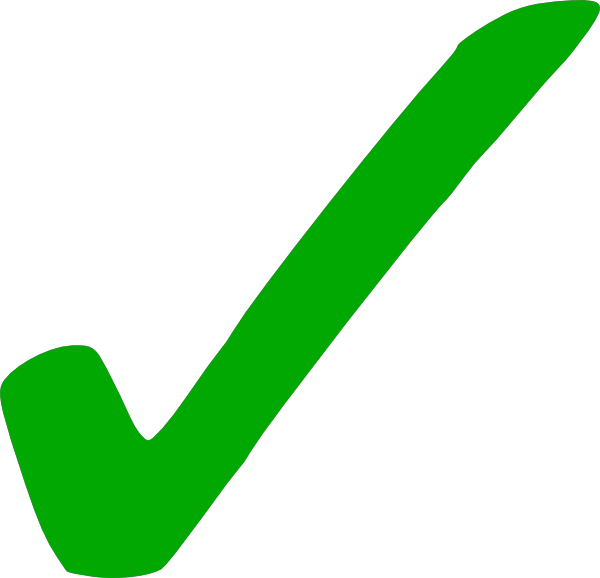 | 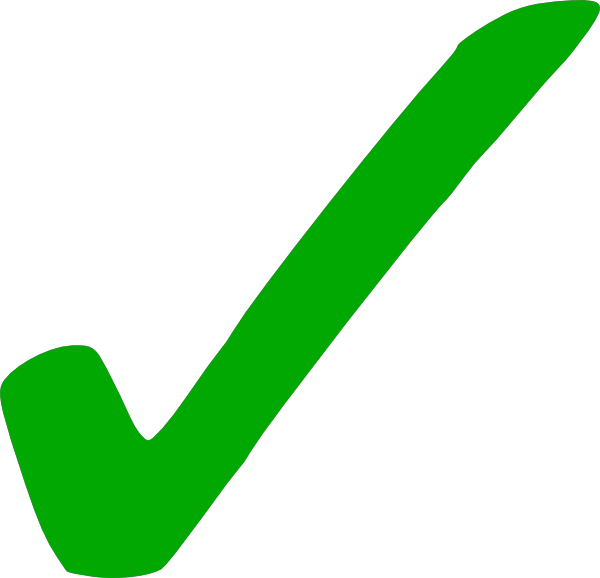 | ❌ | ❌ | ❌ | 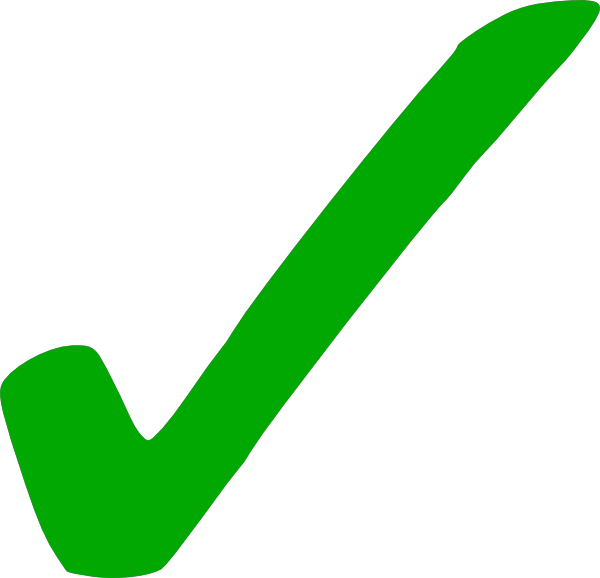 | ❌ | 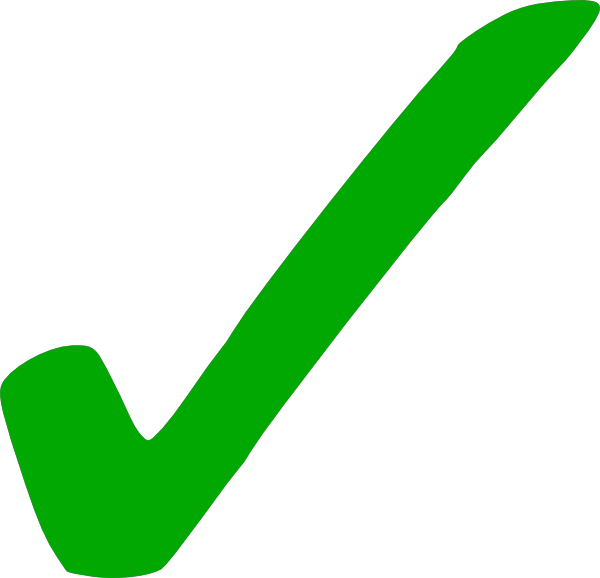 | ❌ | 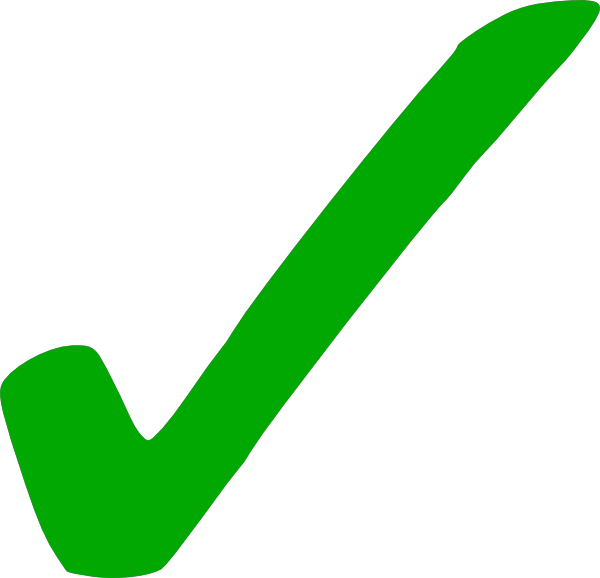 | | 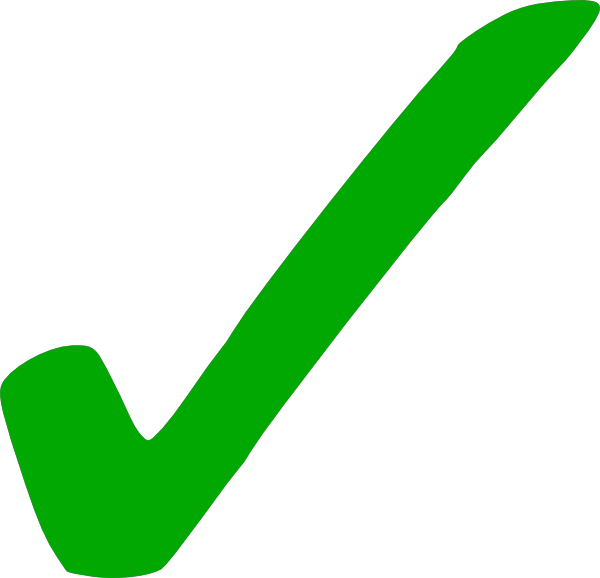 | 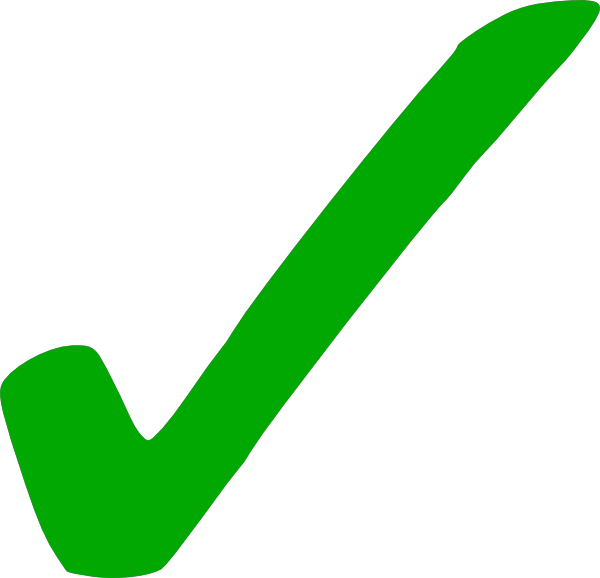 | 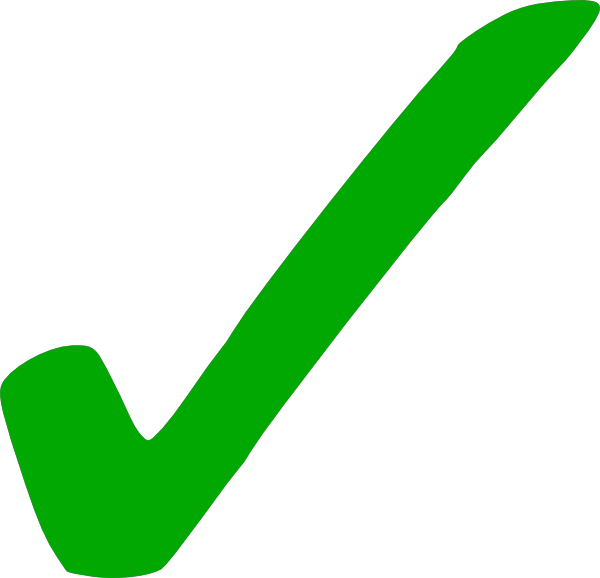 |
| Simonsen et al | 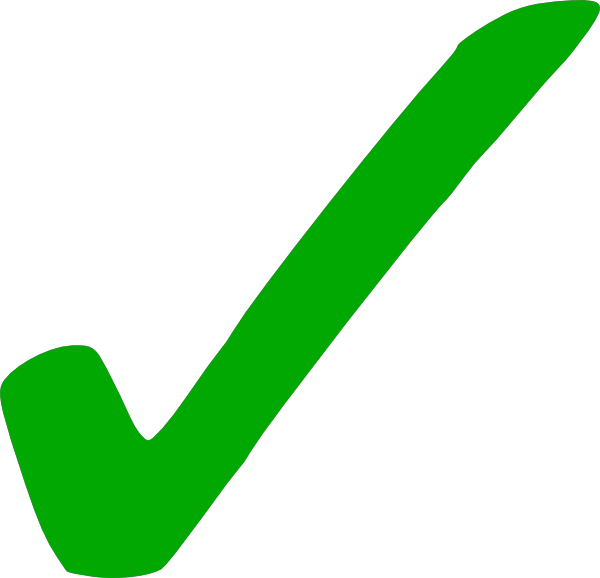 | 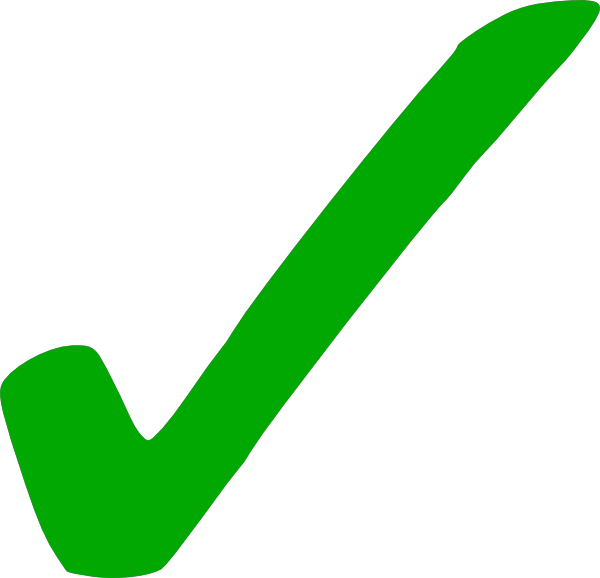 | 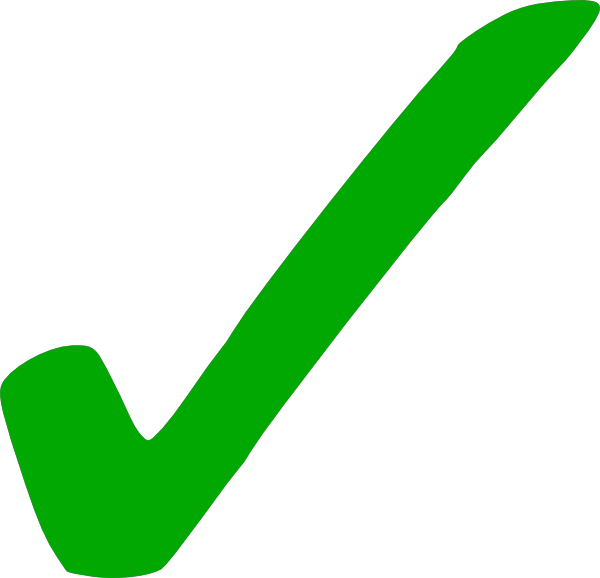 | 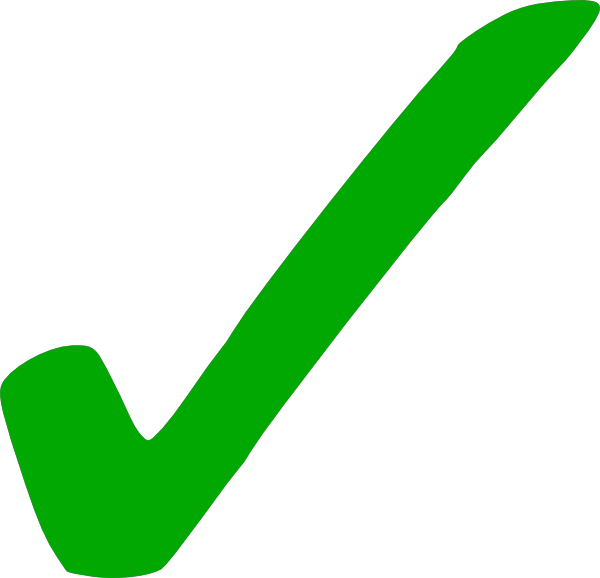 | +/- | +/- | +/- | +/- | 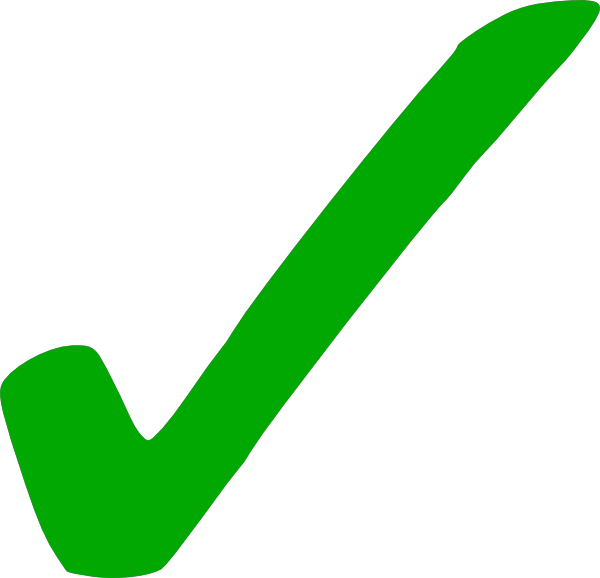 | ❌ | 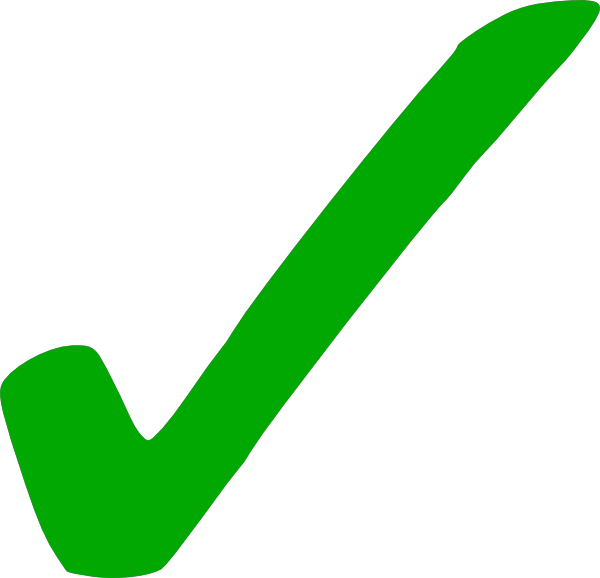 | 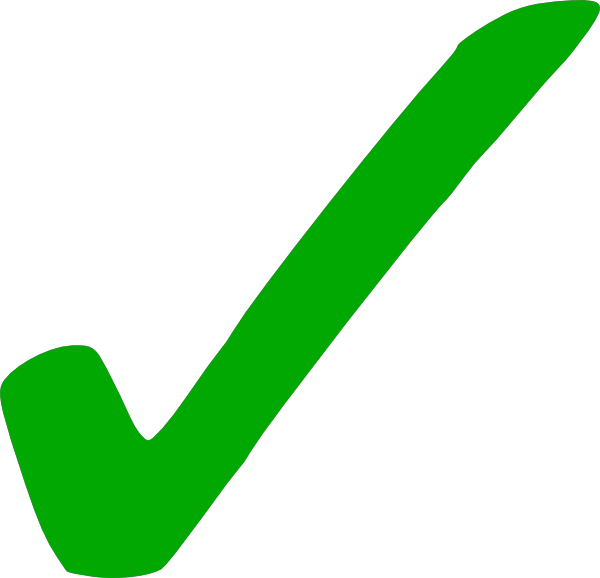 | 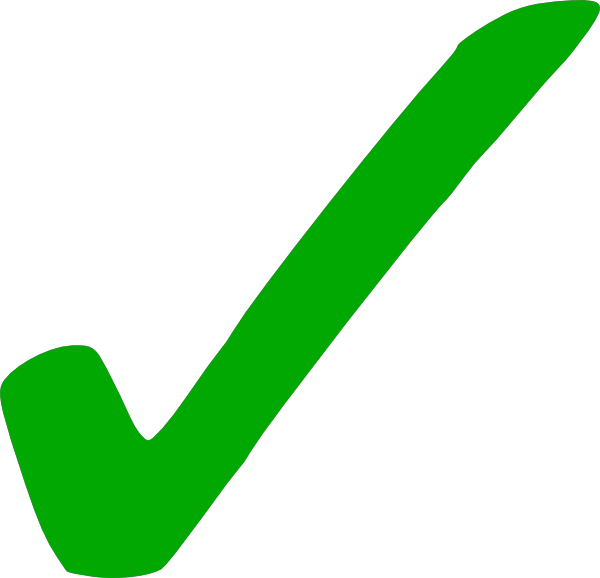 | | 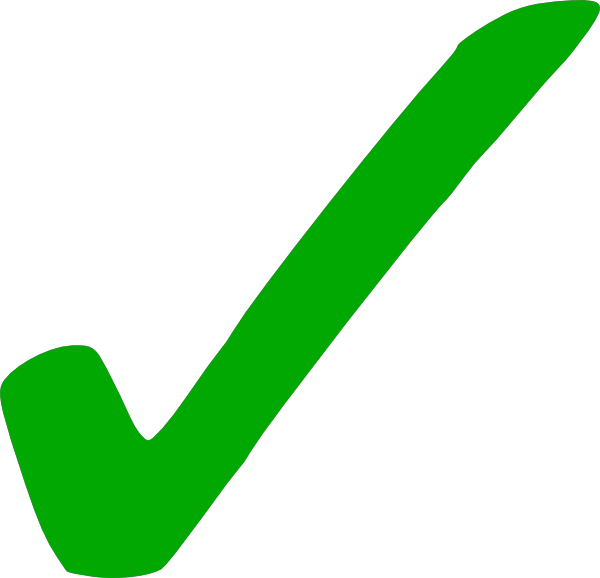 | 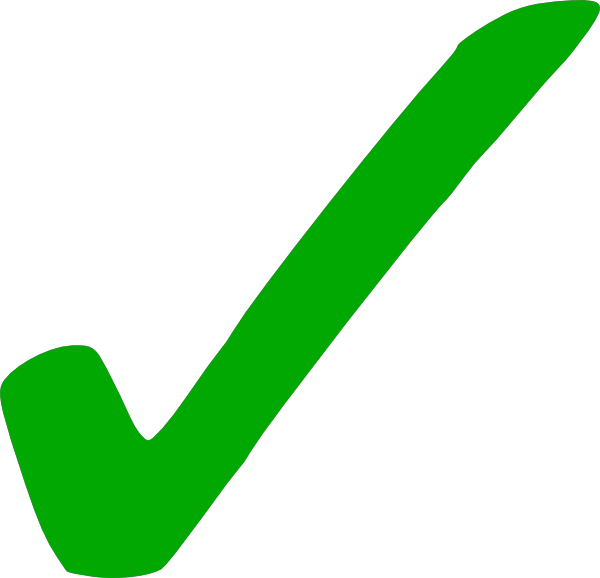 | 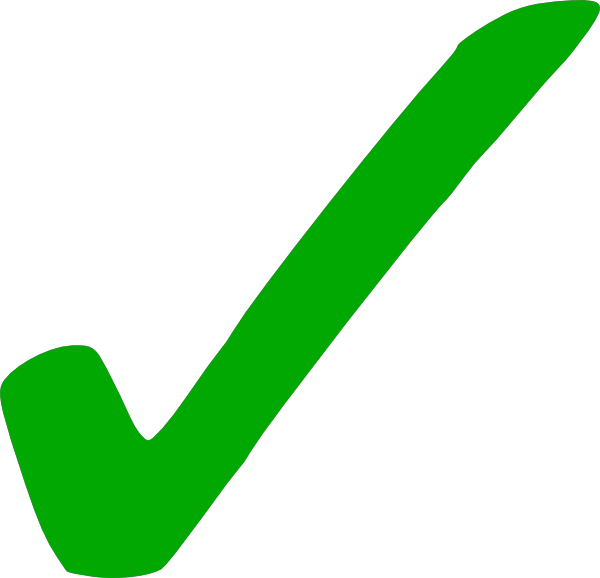 |
| Janak et al | 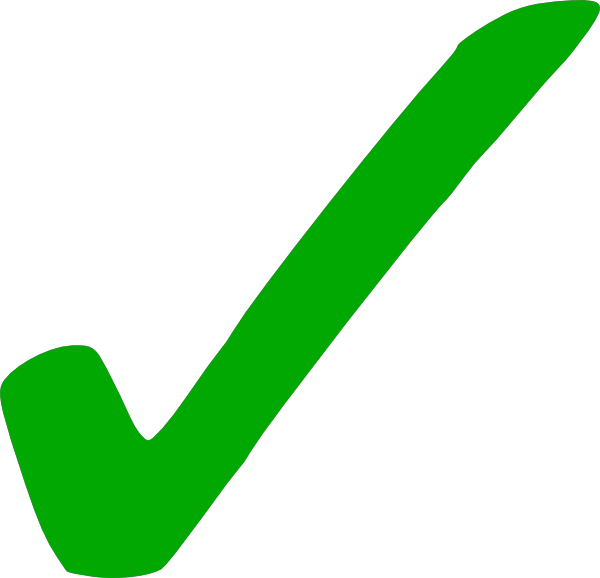 | 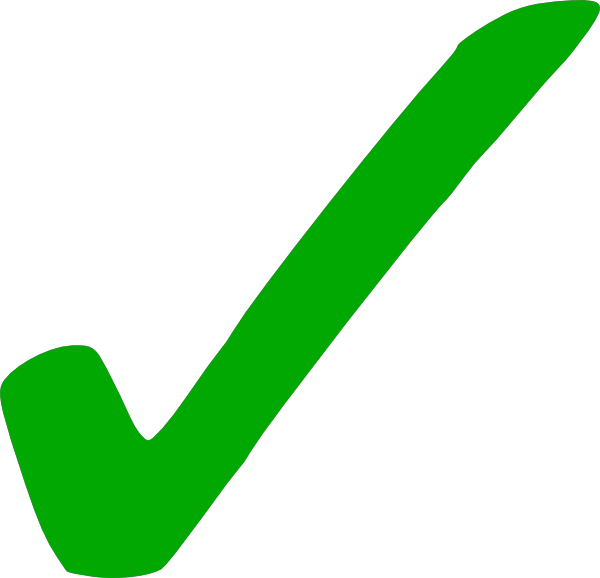 | ❌ | 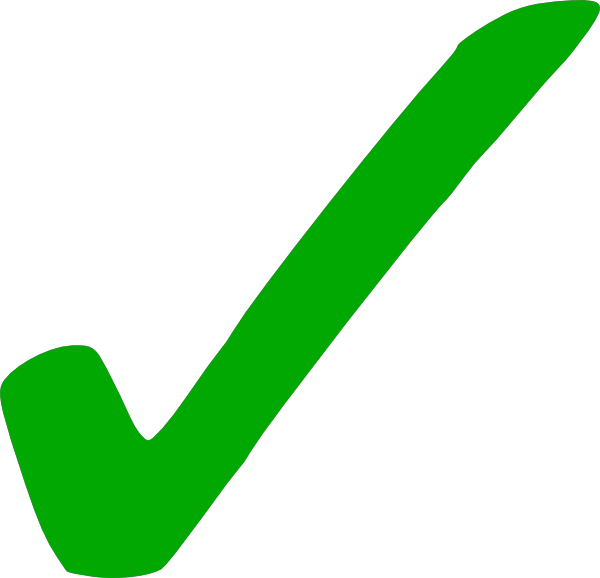 | 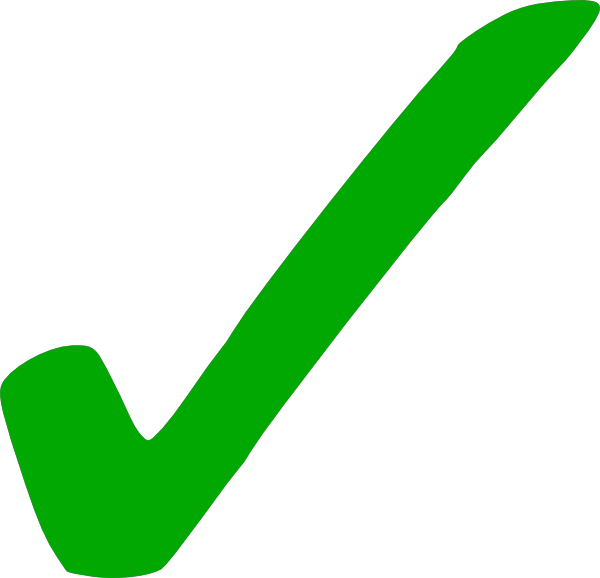 | ❌ | ❌ | ❌ | 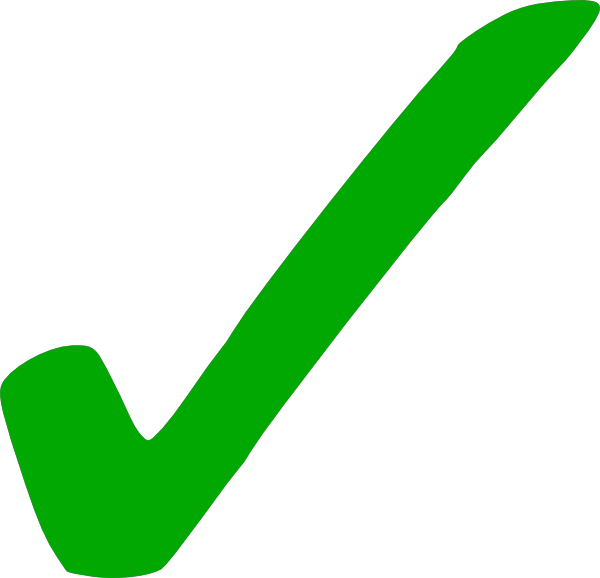 | ❌ | 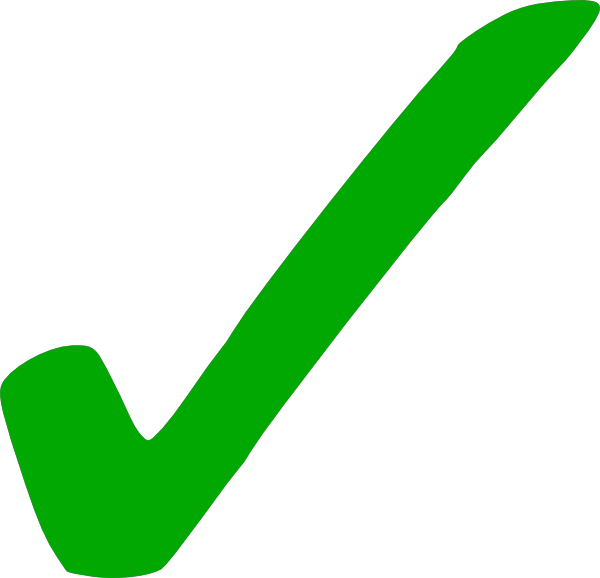 | ❌ | N/A | | | +/- | 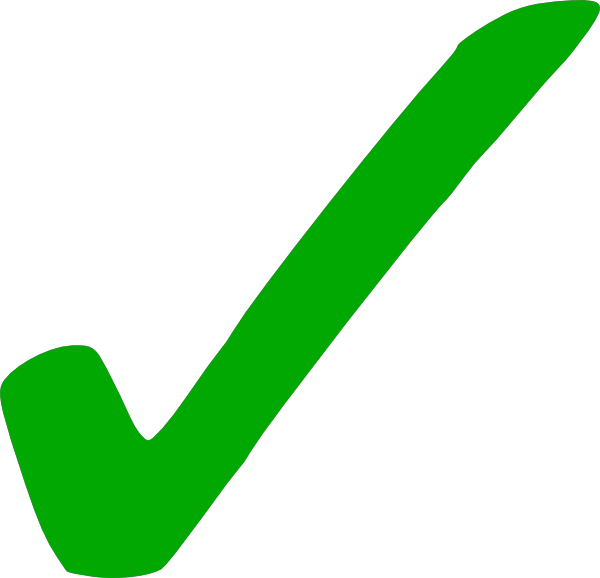 |
| Vanhuyse et al | 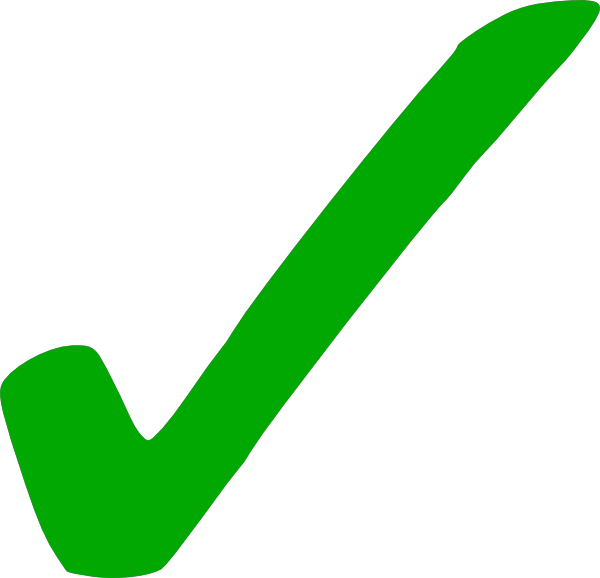 | 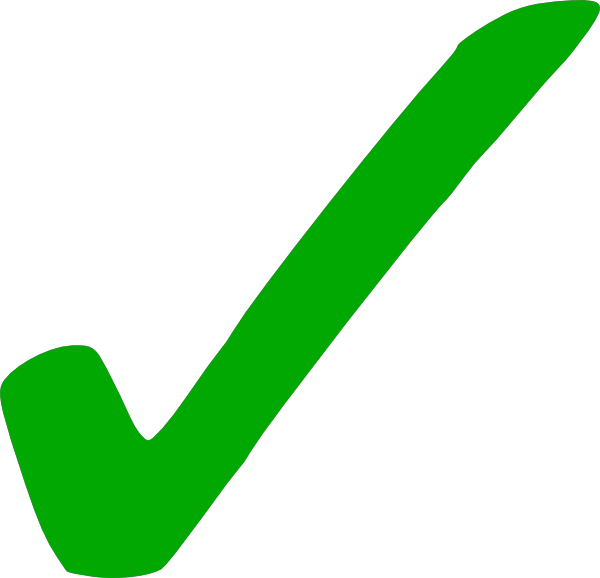 | 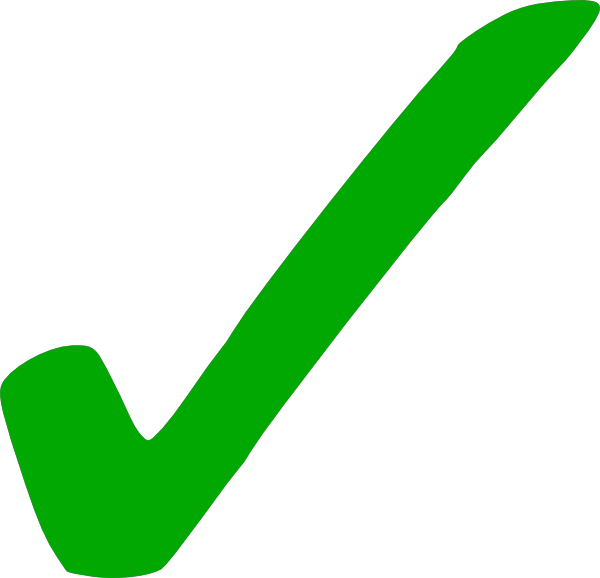 | 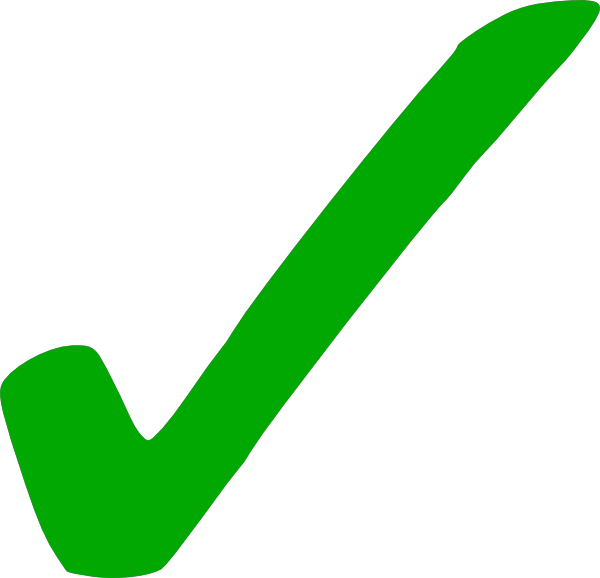 | 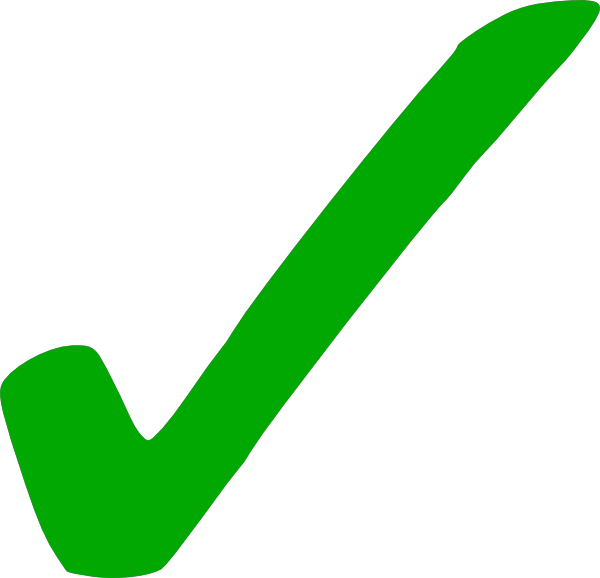 | ❌ | ❌ | ❌ | ❌ | ❌ | 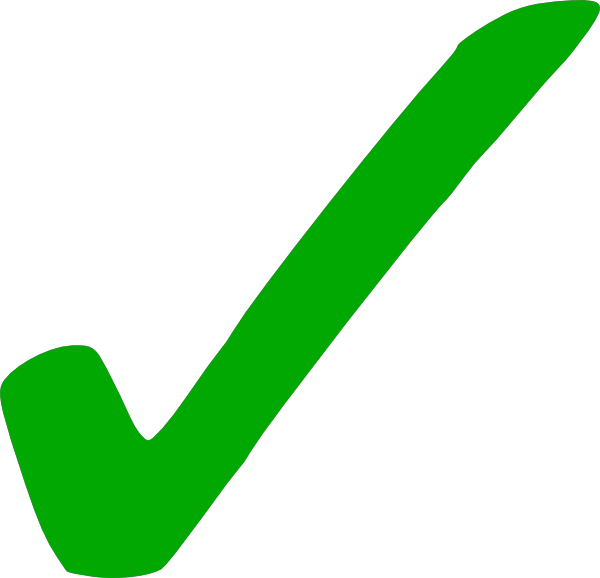 | ❌ | 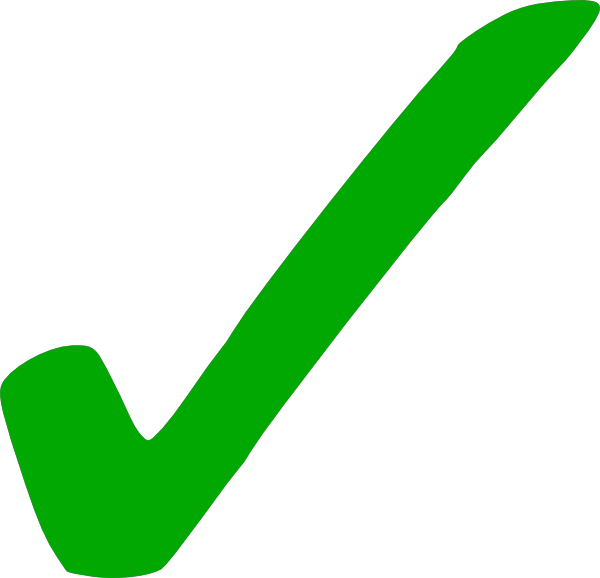 | | 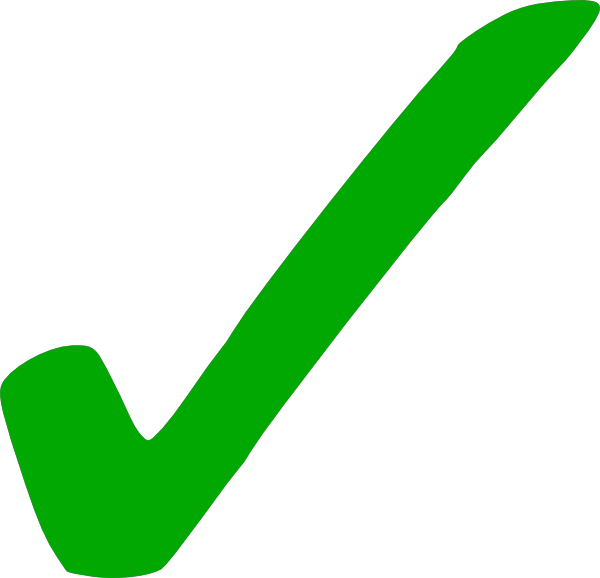 | 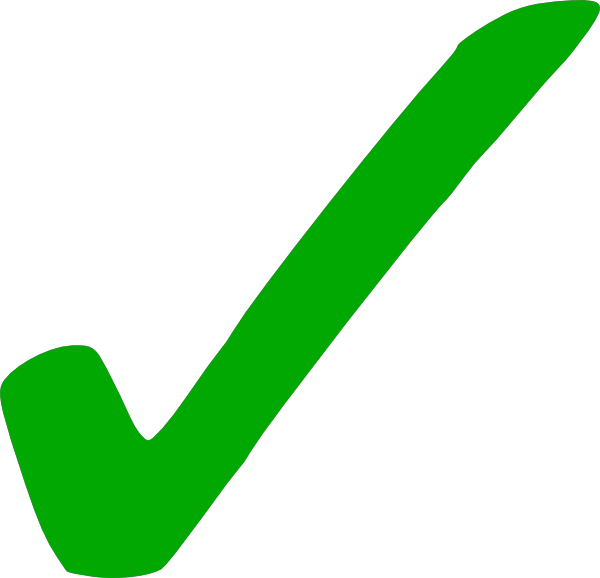 | 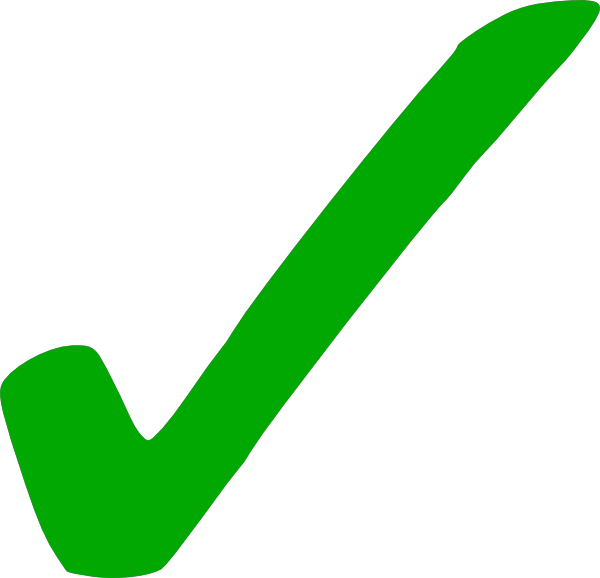 |
| Esposito et al | 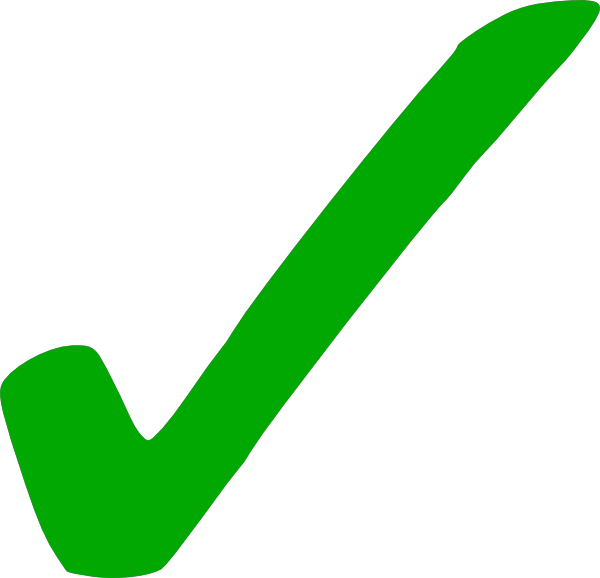 | 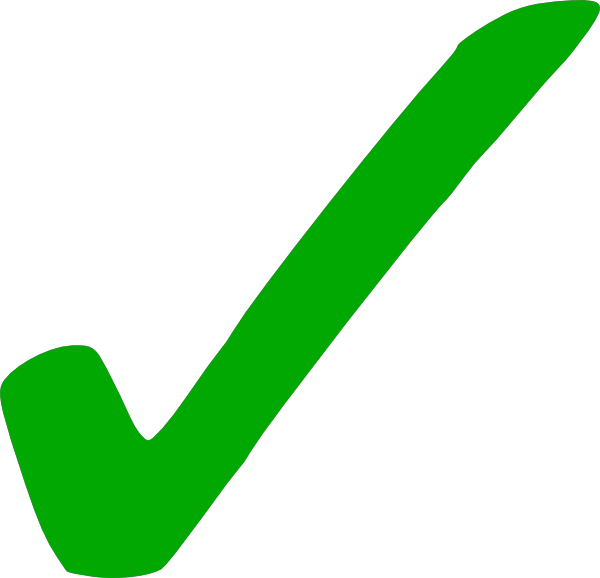 | ❌ | 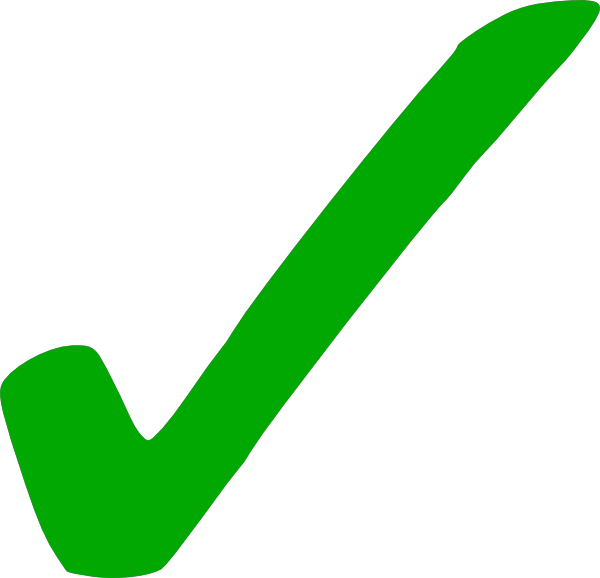 | 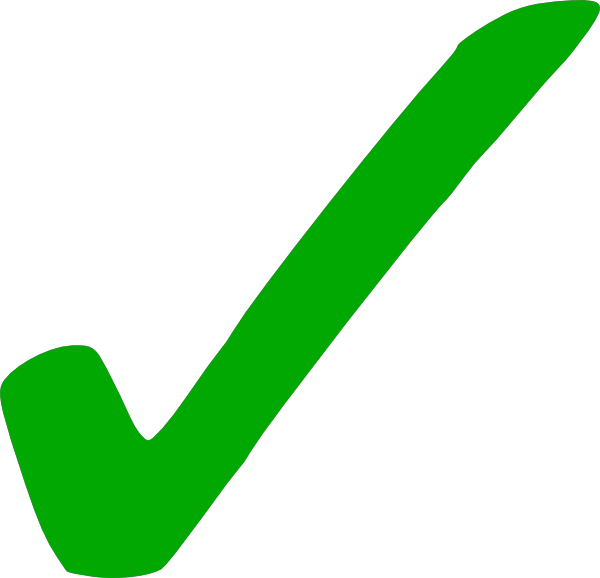 | ❌ | ❌ | ❌ | ❌ | ❌ | 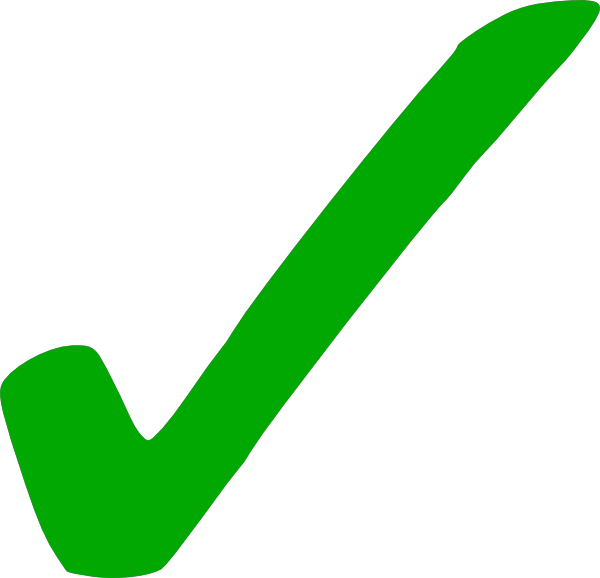 | ❌ | ❌ | | 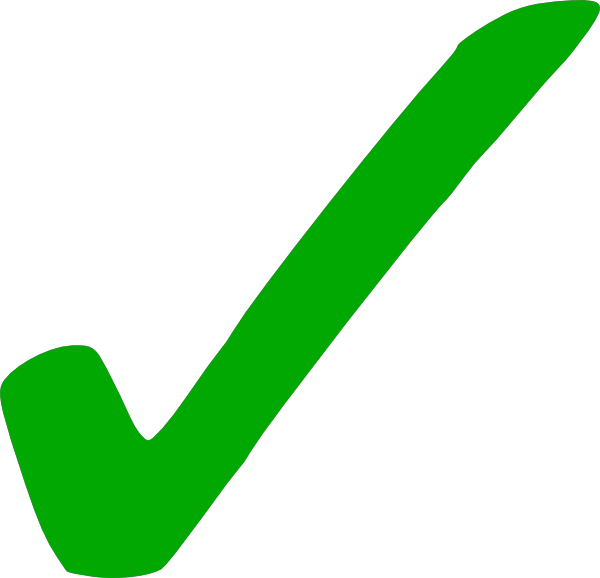 | 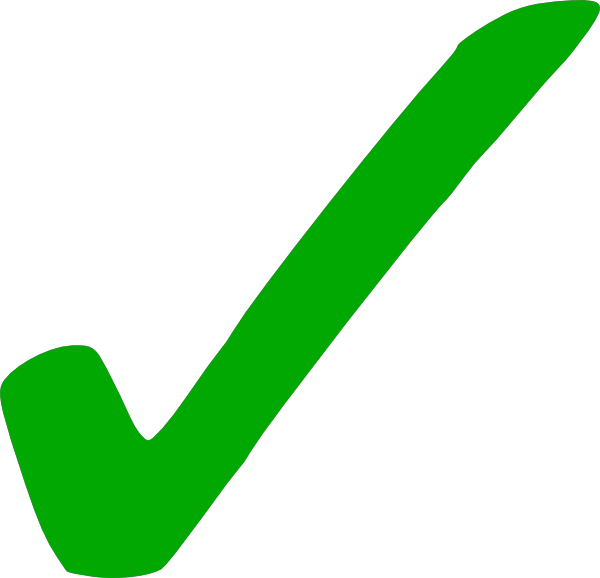 | 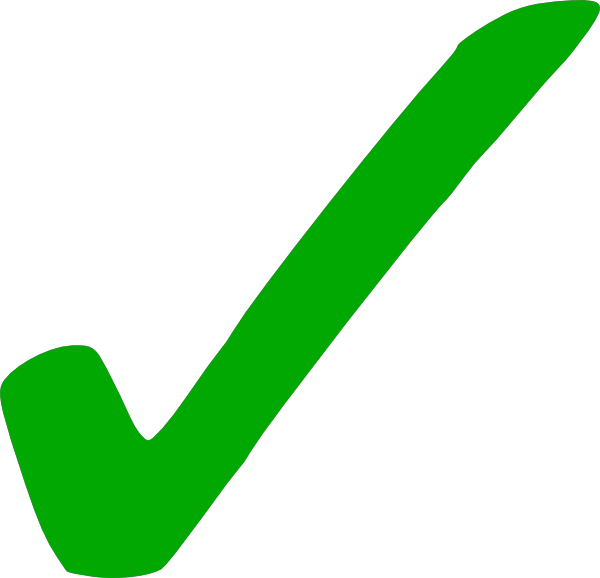 |
| Hala et al | 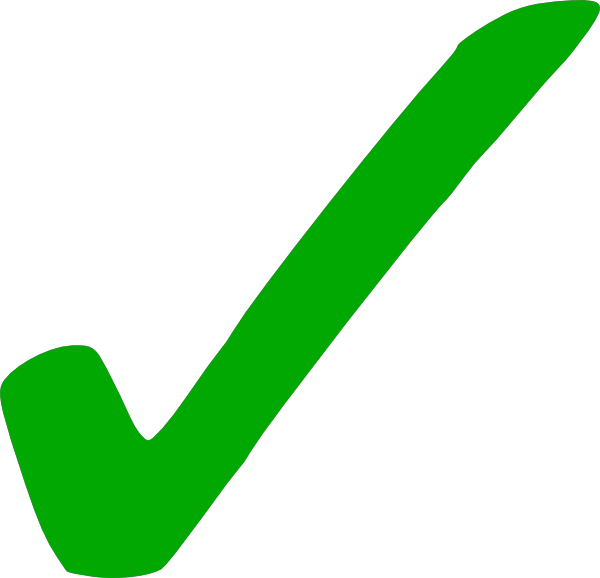 | 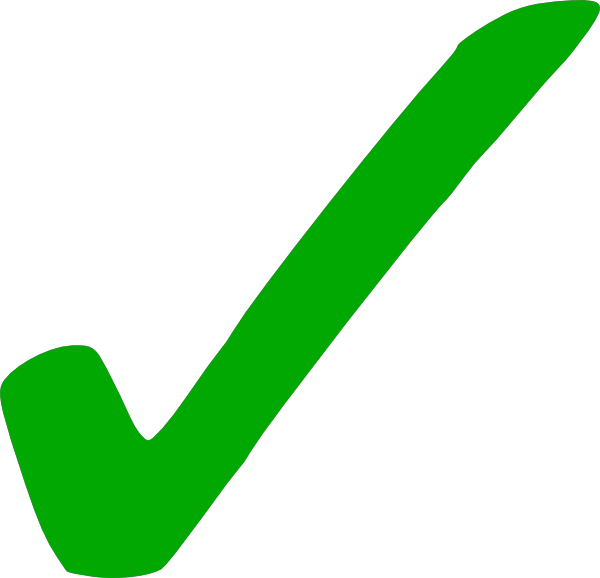 | ❌ | 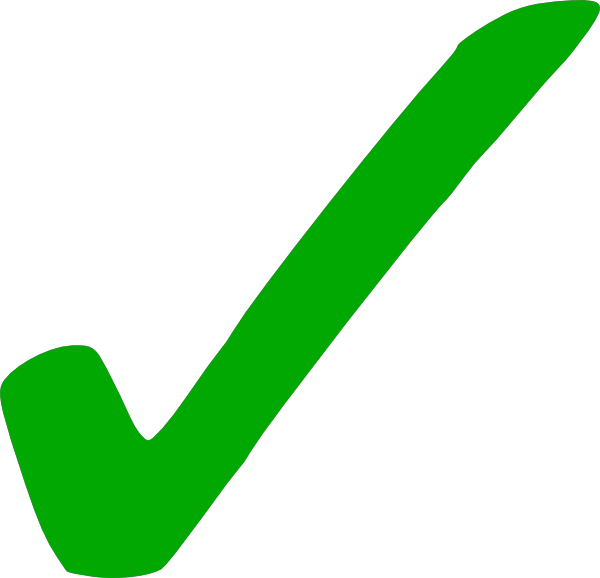 | 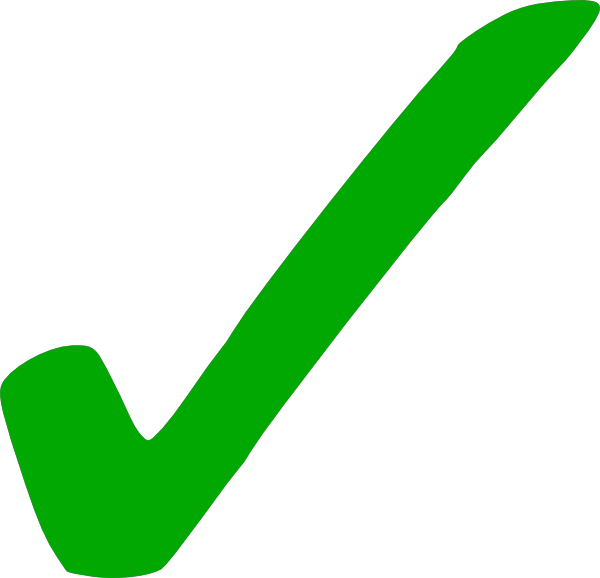 | ❌ | ❌ | ❌ | 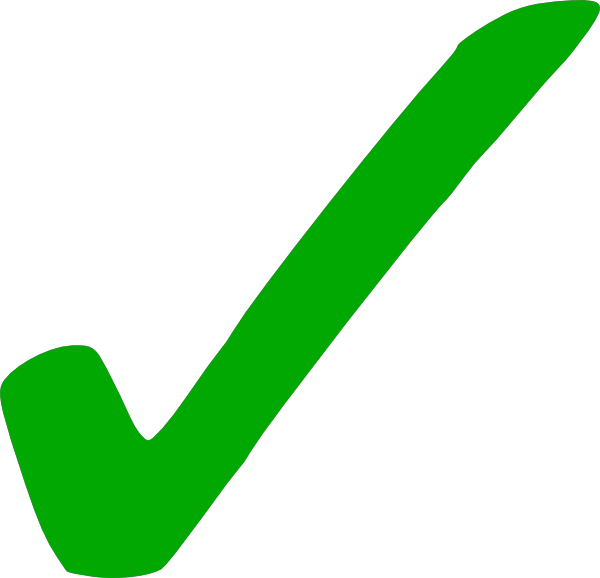 | ❌ | 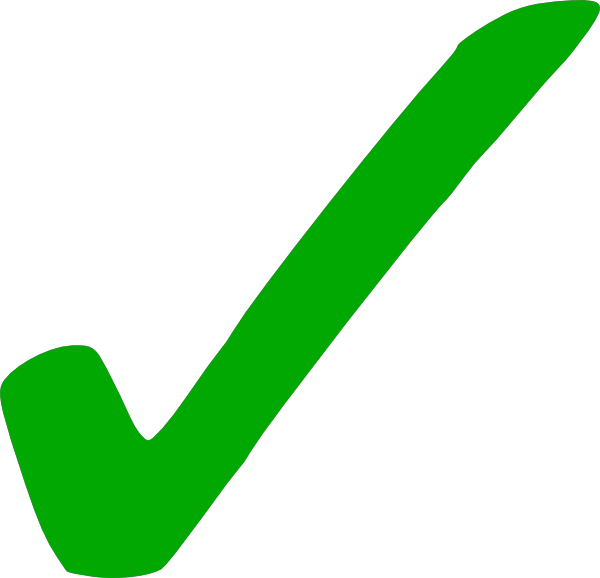 | ❌ | N/A | | | +/- | 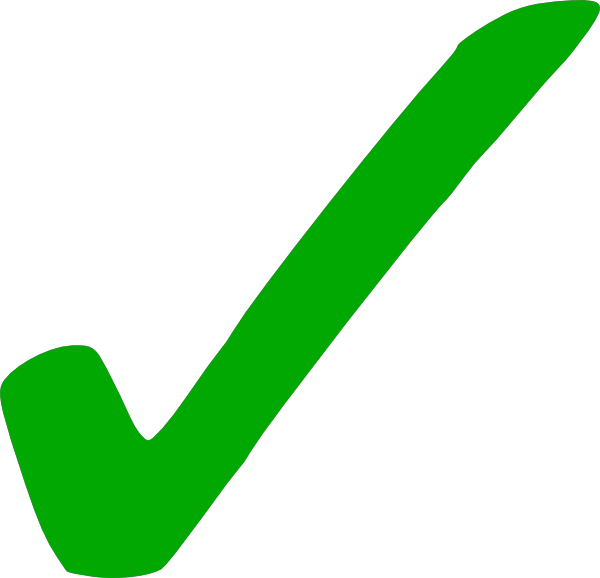 |
| Itoh et al | 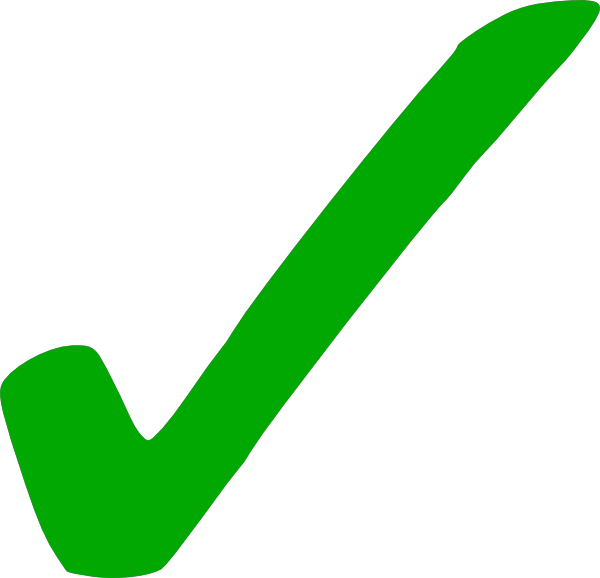 | 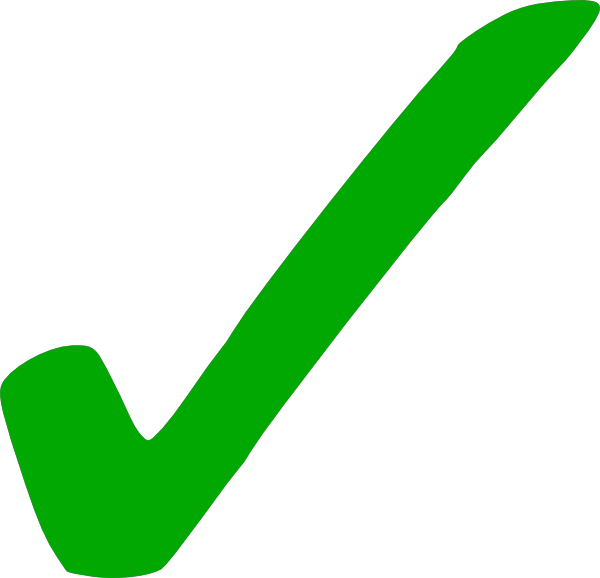 | ❌ | 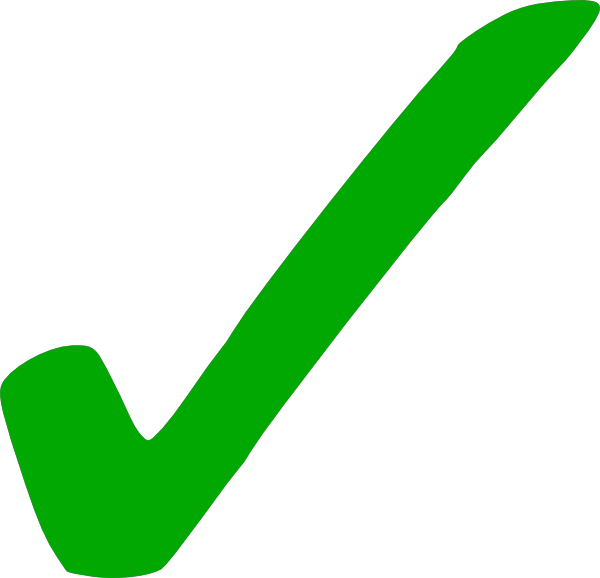 | 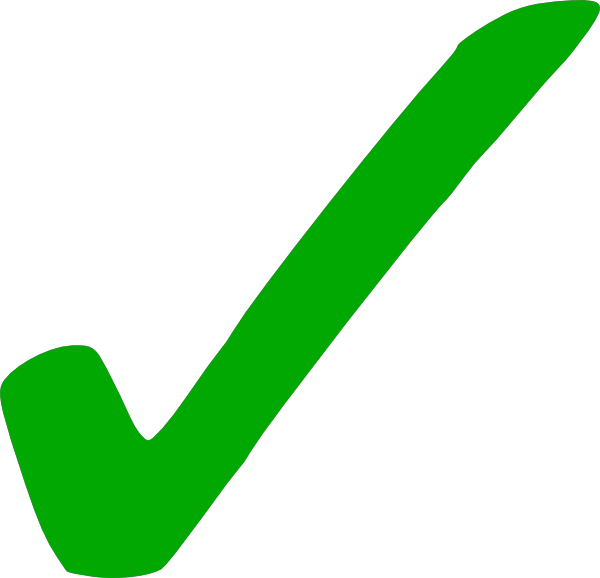 | ❌ | ❌ | ❌ | ❌ | ❌ | 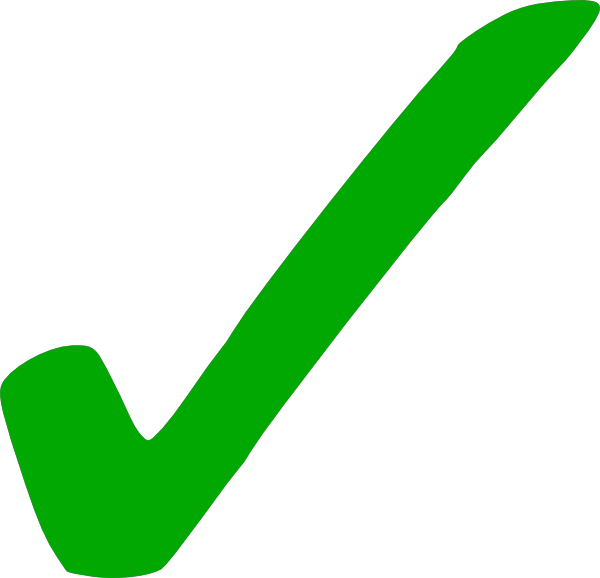 | ❌ | ❌ | | 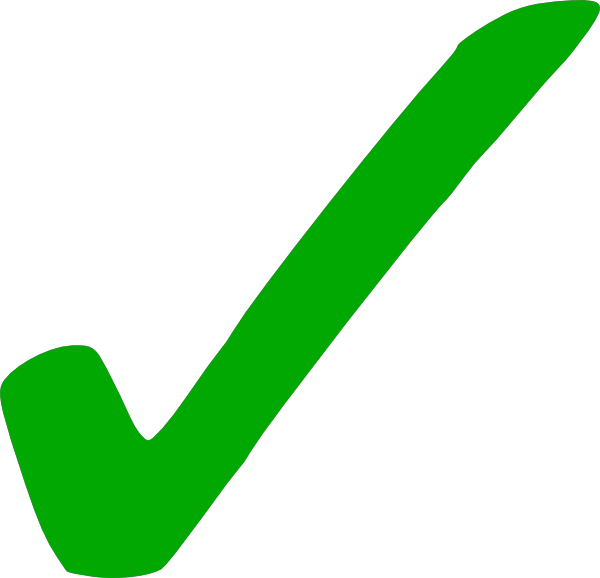 | +/- | 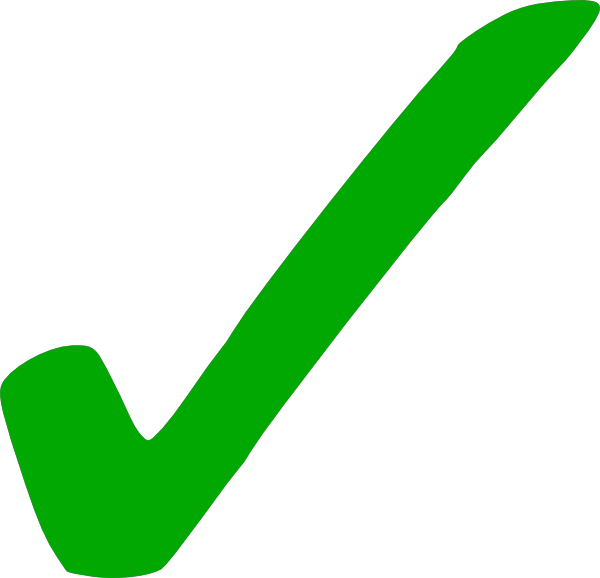 |
| Ostadal et al | 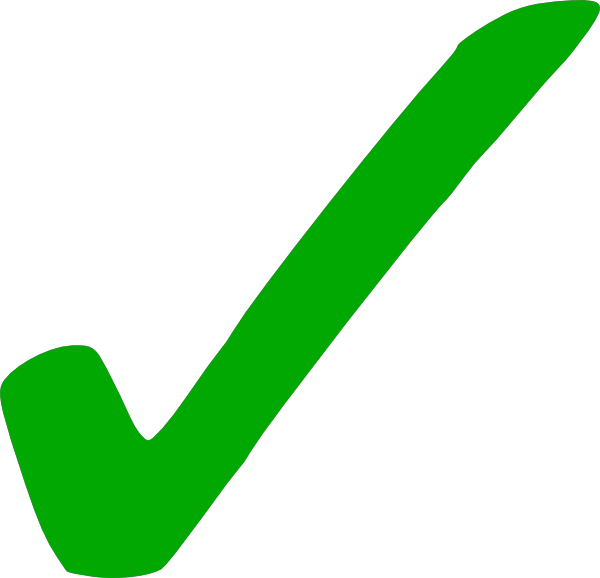 | 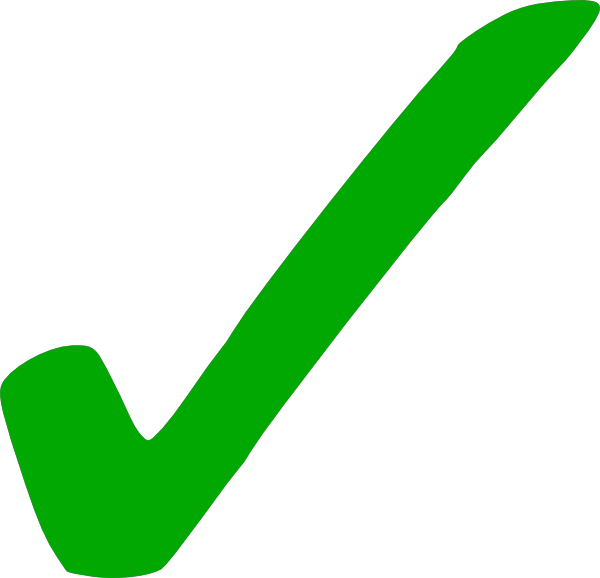 | ❌ | 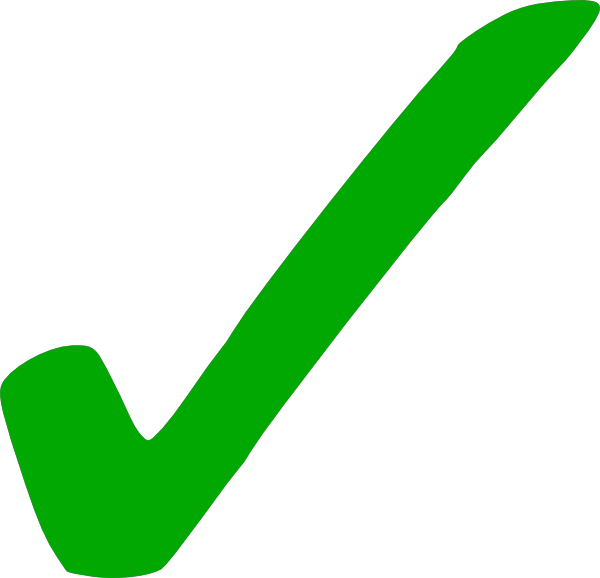 | 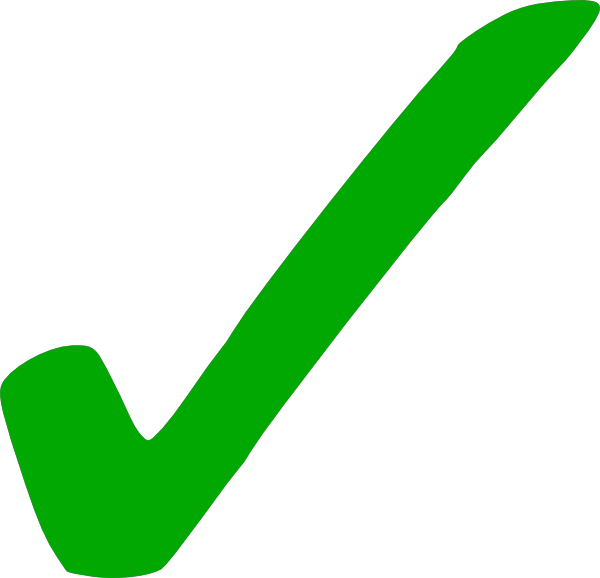 | ❌ | ❌ | ❌ | 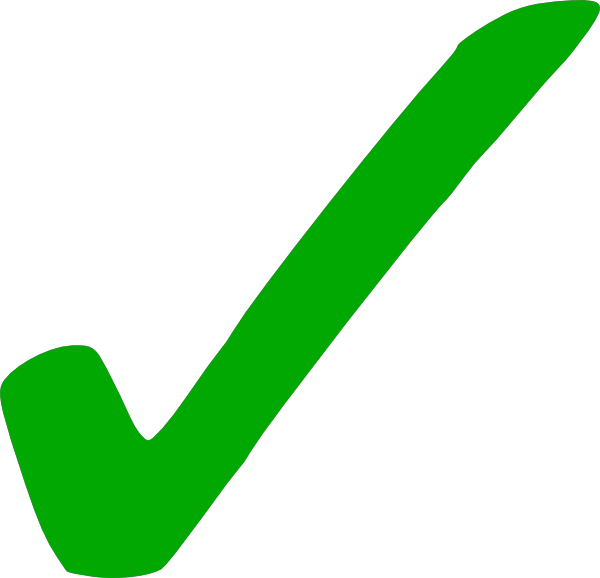 | ❌ | 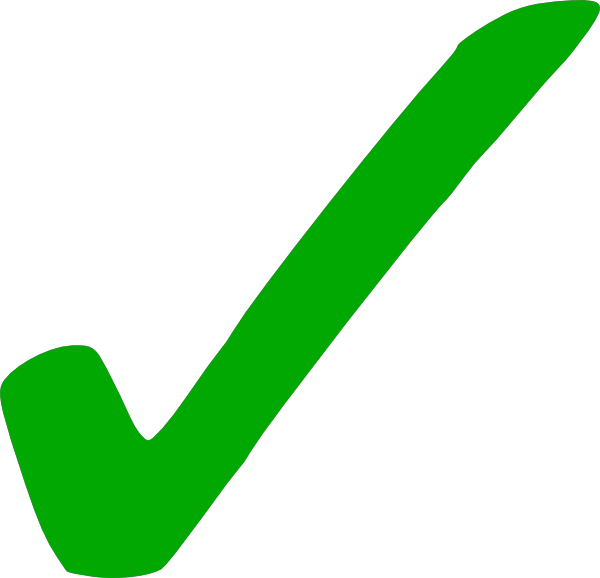 | ❌ | N/A | | | +/- | +/- |
| Brehm et al |  |  | ❌ |  | ❌ | ❌ |  | ❌ | ❌ | ❌ |  | ❌ | N/A | | |  |  |
| Kajimoto et al | +/- |  |  |  |  |  | ❌ | ❌ |  | ❌ |  | ❌ | ❌ | |  |  |  |
| Zhu et al |  |  | ❌ |  |  | ❌ | ❌ | ❌ |  | ❌ |  | ❌ | ❌ | |  | +/- |  |
| Bartoli et al |  |  | ❌ |  |  | ❌ | ❌ | ❌ | ❌ | ❌ |  | ❌ | ❌ | | ❌ |  |  |
| Sauren et al |  |  | ❌ |  |  | ❌ | ❌ | ❌ | +/- | ❌ |  | ❌ | N/A | | | +/- |  |
| Naito et al |  |  | ❌ |  |  | ❌ | ❌ | ❌ | +/- | ❌ |  | ❌ | N/A | | | +/- |  |

*Legends*: = reported; ❌= not reported; +/- = limited reporting.

**eTable 3: Assessment of results according to ARRIVE guidelines checklist [1]**

| **Study** | **Baseline data** | **Numbers analyzed** | | **Outcomes and estimation** | **Adverse events** | |
| --- | --- | --- | --- | --- | --- | --- |
|  |  | **Absolute numbers** | **Exclusion reason** |  | **Important AE** | **Modification of protocol to reduce AE** |
| Sakamoto et al |  |  | N/A |  | ❌ | ❌ |
| Kawashima et al |  |  |  |  |  | ❌ |
| Yu et al |  |  | N/A |  | ❌ | ❌ |
| Segesser et al | ❌ |  | N/A |  | ❌ | ❌ |
| Møller-Helgestad et al |  |  |  |  |  | ❌ |
| Ostadal et al |  |  | N/A |  | ❌ | ❌ |
| Simonsen et al |  |  |  |  |  |  |
| Janak et al | ❌ |  | N/A |  | ❌ | ❌ |
| Vanhuyse et al |  |  | N/A |  | ❌ | ❌ |
| Esposito et al |  |  |  |  |  | ❌ |
| Hala et al | ❌ |  | N/A |  | ❌ | ❌ |
| Itoh et al |  |  | N/A |  | ❌ | ❌ |
| Ostadal et al | ❌ |  | N/A |  |  | ❌ |
| Brehm et al |  |  | N/A |  | ❌ | ❌ |
| Kajimoto et al |  |  |  |  |  | ❌ |
| Zhu et al | +/- |  | ❌ |  | ❌ | ❌ |
| Bartoli et al |  |  |  |  |  | ❌ |
| Sauren et al |  |  |  |  |  | ❌ |
| Naito et al |  |  | N/A |  | ❌ | ❌ |

*Legends*: = reported; ❌= not reported; N/A= non-applicable

[1] Kilkenny C, Browne W, Cuthill IC, Emerson M, Altman DG, Group NCRRGW, (2010) Animal research: reporting in vivo experiments: the ARRIVE guidelines. Br J Pharmacol 160: 1577-1579

**eTable 4:** **anaesthetic and ventilatory management pre-cardiac failure induction**

| Variables, n(%) | All studies (n=19) |
| --- | --- |
| Animals fasted  Yes  No  Not reported | 6  0  13 |
| Pre-medication  Yes  Not reported | 12 (63)  7 (37) |
| Induction of anaesthesia |  |
| Intravenous  Propofol + opioid (morphine or sufentanyl)  Thiopenthal  Propofol | 8 (42)  4  3  2 |
| Intramuscular | 3 (16) |
| Inhalation (isoflurane) | 2 (11) |
| Combination (intramuscular + intraperitoneal) | 1 (5) |
| Not reported | 5 (26) |
| Maintenance of anaesthesia |  |
| Inhalation  Isoflurane  Isoflurane + N2O  N2O  Not reported | 8 (42)  6  1  1  1 |
| Intravenous  Propofol  Propofol + Midazolam  Propofol + opioid (morphine or remifentanil or sufentanyl)  Midazolam + Fentanyl + Ketamine | 9 (47)  1  2  5  1 |
| Not reported | 1 (5) |
| Airway management  Endotracheal tube  Tracheostomy  Not reported | 13 (68)  3 (16)  3 (16) |

**PRISMA 2009 checklist [2]**

| **Section/topic** | **#** | **Checklist item** | **Reported on page #** |
| --- | --- | --- | --- |
| **TITLE** | | |  |
| Title | 1 | Identify the report as a systematic review, meta-analysis, or both. | 1 |
| **ABSTRACT** | | |  |
| Structured summary | 2 | Provide a structured summary including, as applicable: background; objectives; data sources; study eligibility criteria, participants, and interventions; study appraisal and synthesis methods; results; limitations; conclusions and implications of key findings; systematic review registration number. | 2 |
| **INTRODUCTION** | | |  |
| Rationale | 3 | Describe the rationale for the review in the context of what is already known. | 3 |
| Objectives | 4 | Provide an explicit statement of questions being addressed with reference to participants, interventions, comparisons, outcomes, and study design (PICOS). | 4 |
| **METHODS** | | |  |
| Protocol and registration | 5 | Indicate if a review protocol exists, if and where it can be accessed (e.g., Web address), and, if available, provide registration information including registration number. | 4 |
| Eligibility criteria | 6 | Specify study characteristics (e.g., PICOS, length of follow-up) and report characteristics (e.g., years considered, language, publication status) used as criteria for eligibility, giving rationale. | 4 and 5 |
| Information sources | 7 | Describe all information sources (e.g., databases with dates of coverage, contact with study authors to identify additional studies) in the search and date last searched. | 4 |
| Search | 8 | Present full electronic search strategy for at least one database, including any limits used, such that it could be repeated. | ESM |
| Study selection | 9 | State the process for selecting studies (i.e., screening, eligibility, included in systematic review, and, if applicable, included in the meta-analysis). | 4, 5 |
| Data collection process | 10 | Describe method of data extraction from reports (e.g., piloted forms, independently, in duplicate) and any processes for obtaining and confirming data from investigators. | 5 |
| Data items | 11 | List and define all variables for which data were sought (e.g., PICOS, funding sources) and any assumptions and simplifications made. | ESM |
| Risk of bias in individual studies | 12 | Describe methods used for assessing risk of bias of individual studies (including specification of whether this was done at the study or outcome level), and how this information is to be used in any data synthesis. | No assessment |
| Summary measures | 13 | State the principal summary measures (e.g., risk ratio, difference in means). | 6 |
| Synthesis of results | 14 | Describe the methods of handling data and combining results of studies, if done, including measures of consistency (e.g., I^2^) for each meta-analysis. | N/A |
| Risk of bias across studies | 15 | Specify any assessment of risk of bias that may affect the cumulative evidence (e.g., publication bias, selective reporting within studies). | No assessment |
| Additional analyses | 16 | Describe methods of additional analyses (e.g., sensitivity or subgroup analyses, meta-regression), if done, indicating which were pre-specified. | N/A |
| **RESULTS** | | |  |
| Study selection | 17 | Give numbers of studies screened, assessed for eligibility, and included in the review, with reasons for exclusions at each stage, ideally with a flow diagram. | 6 + tables and figures |
| Study characteristics | 18 | For each study, present characteristics for which data were extracted (e.g., study size, PICOS, follow-up period) and provide the citations. | Tables and figures |
| Risk of bias within studies | 19 | Present data on risk of bias of each study and, if available, any outcome level assessment (see item 12). | N/A |
| Results of individual studies | 20 | For all outcomes considered (benefits or harms), present, for each study: (a) simple summary data for each intervention group (b) effect estimates and confidence intervals, ideally with a forest plot. | Tables and figures |
| Synthesis of results | 21 | Present results of each meta-analysis done, including confidence intervals and measures of consistency. | N/A |
| Risk of bias across studies | 22 | Present results of any assessment of risk of bias across studies (see Item 15). | No assessment |
| Additional analysis | 23 | Give results of additional analyses, if done (e.g., sensitivity or subgroup analyses, meta-regression [see Item 16]). | N/A |
| **DISCUSSION** | | |  |
| Summary of evidence | 24 | Summarize the main findings including the strength of evidence for each main outcome; consider their relevance to key groups (e.g., healthcare providers, users, and policy makers). | 6 to 8 |
| Limitations | 25 | Discuss limitations at study and outcome level (e.g., risk of bias), and at review-level (e.g., incomplete retrieval of identified research, reporting bias). | 9 to 11 |
| Conclusions | 26 | Provide a general interpretation of the results in the context of other evidence, and implications for future research. | 8 to 12 |
| **FUNDING** | | |  |
| Funding | 27 | Describe sources of funding for the systematic review and other support (e.g., supply of data); role of funders for the systematic review. | 12 |

**[2]** Moher D, Liberati A, Tetzlaff J, Altman DG, The PRISMA Group (2009). Preferred Reporting Items for Systematic Reviews and Meta-Analyses: The PRISMA Statement. PLoS Med 6(7): e1000097. doi:10.1371/journal.pmed1000097
